# Redefining the Geographic Distribution of Two Cryptic *Halictus* (Hymenoptera: Halictidae) Species in the Eastern United States

**DOI:** 10.1002/ece3.71570

**Published:** 2025-06-20

**Authors:** Hannah K. Levenson, Luke R. Tembrock, Frida A. Zink, Kayla A. Mollet, David R. Tarpy

**Affiliations:** ^1^ Department of Entomology and Plant Pathology North Carolina State University Raleigh North Carolina USA; ^2^ Department of Agricultural Biology Colorado State University Fort Collins Colorado USA; ^3^ Department of Applied Ecology North Carolina State University Raleigh North Carolina USA

**Keywords:** CO1 barcoding, cryptic species, molecular identification, native bees, primer optimization

## Abstract

Incomplete characterization of cryptic species complexes in pollinator communities can limit our understanding of ecosystem function, population dynamics, effects of environmental perturbations, and conservation planning. Molecular tools to distinguish morphologically indistinguishable bee species are therefore necessary but require refinement and validation to make robust inferences. Here we present newly developed primers and demonstrate their successful use for identification of two cryptic bee species, *Halictus ligatus* and *Halictus poeyi* , with overlapping ranges in the mid‐Atlantic USA. We found that *H. ligatus* is present at higher elevations while *H. poeyi* is present at lower elevations, with both species present at three sample sites in central North Carolina, USA. The data generated in this study was combined with publicly available sequence data and analyzed to make inferences about the species ranges of these two bees in the Western Hemisphere. These clarified species distributions help us better understand local pollinator communities, associated habitat features, and abiotic conditions amenable to each, as well as provide insights into patterns related to their speciation.

## Graphical abstract

Molecular tools to distinguish morphologically identical bee species are necessary but require refinement and validation to make robust inferences. Here we present newly developed primers and demonstrate their successful use for identification of two cryptic bee species, *Halictus ligatus* and *Halictus poeyi* . Clarified species distributions help us better understand local pollinator communities, associated habitat features, and abiotic conditions amenable to each, as well as provide insights into patterns related to their speciation.

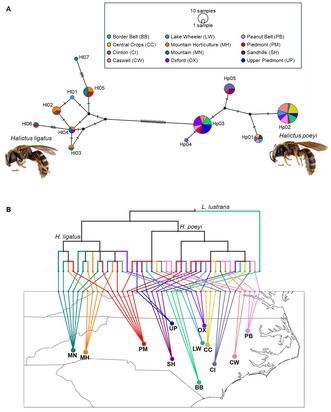

## 1 Introduction

Cryptic species—or those that were originally, erroneously classified as a single species, typically through morphological identification, that are later reclassified as multiple species when reexamined with additional data—have been documented across many geographic areas and numerous branches of life (Bickford et al. 2006; Struck et al. 2018). The presence of cryptic species can have significant consequences for our understanding of biodiversity, biogeographic patterns, ecosystem functioning, niche partitioning, and conservation planning (Bickford et al. 2006; Struck et al. 2018; Suatoni et al. 2006) as their misidentification as a single species obscures the proper association of a trait with a phylogenetically defined lineage. Thus, the identification of cryptic species is of great interest to aid in understanding how phylogeny is associated with patterns in geographic distribution, behavior, and ecological adaptation. The introduction of molecular identification techniques has greatly improved the discovery and diagnosis of cryptic species, although challenges still remain (Horsáková et al. 2019; Jörger and Schrödl 2013).

One ecologically and economically important lineage of insects that contains several well‐known examples of cryptic species complexes is bees, particularly halictid bees (Packer and Taylor 1997). It has been predicted that the true number of bee species is greatly underestimated due to the existence of unrecognized, cryptic species (Packer and Taylor 1997). Several bee taxa are notorious for being difficult to identify using morphology—such as *Lasiolgossum* (Gibbs 2009, 2012; Gardner and Gibbs 2023) and green sweat bees (Portman et al. 2022, 2024)—and thus, are likely to contain undescribed species. In many of these cases, morphological approaches fail to reliably distinguish between cryptic species (Packer et al. 2009), but as molecular tools for bee research are developed (Schmidt et al. 2015), research involving cryptic species complexes has advanced (Gibbs et al. 2012; Landaverde‐González et al. 2017; Mayr et al. 2021).

One noteworthy example of cryptic bee species is *Halictus ligatus* and *H. poeyi* . *Halictus ligatus* , through morphological descriptions, was once thought to be one highly variable species (Mitchell 1960), common across all of Eastern North America (Michener and Bennett 1977). But *H. ligatus* was later discovered to be two cryptic species using allozyme data, with distinct but overlapping ranges (Carman and Packer 1996). This was further confirmed by Danforth et al. (1998) using DNA sequencing. The species in the Southern United States (USA) was reclassified as *H. poeyi*, and the species present throughout the Northern USA, Western USA, and Canada retained the name *H. ligatus* (Carman and Packer 1996). Carman and Packer (1996) based the name *H. poeyi* on a name synonymized with *H. ligatus* , described from Cuba, but suggested that the name *H. capitosus* , also synonymized with *H. ligatus* based on collections from Florida, would have to be applied to the Southeastern lineage if the Cuban and Florida lineages proved to be divergent. Currently, the colloquial range distinction between the two species in the eastern USA is typically *H. ligatus* in the north and *H. poeyi* in the south, with a horizontal line in Virginia as the putative boundary between the two species' ranges; however, previous studies have documented range overlap at three sampling locations: Chattanooga, Tennessee, USA; Rock Hill, South Carolina, USA; and Charlotte, North Carolina (NC), USA (Carman and Packer 1996; Danforth et al. 1998).

Because these two *Halictus* species are morphologically indistinguishable, even when using wing morphometrics or dissection of male genitalia, molecular testing is needed for their definitive identification (Packer et al. 2016). This is critical in helping to resolve areas of sympatry at the species range boundaries, and an effort to do so in this species complex was initially conducted using allozyme data (Carman and Packer 1996; Packer 1999). Yet even today, when specimens in this species complex are collected, they are often identified by inference based on the sampling location. For specimens collected in states along the colloquial border, such as NC, a putative species determination cannot be made, and specimens are regularly listed as *H. ligatus/poeyi* during reporting (see Hamblin et al. 2018; Levenson et al. 2023; Levenson and Tarpy 2022; Ruzi et al. 2023 as examples).

In our efforts, we first attempted to identify *H. ligatus* and *H. poeyi* by DNA sequencing using universal primers to amplify part of the mitochondrial cytochrome c oxidase 1 (CO1) gene (e.g., Simon et al. 1994; Folmer et al. 1994), which was then sequenced for DNA barcoding. DNA barcoding necessarily requires sequencing the same portion of a single gene, typically mitochondrial CO1, for comparison within and between species (Hebert et al. 2003). There are several technical issues that can arise when diagnosing individual specimens to species using DNA barcoding or when defining species through this method (see Rubinoff et al. 2006). Despite these issues, CO1 barcode data are unparalleled as a comparative tool in molecular genetics for species description and discovery in large part because of the amassment of huge CO1 databases available for public use (e.g., Chan et al. 2014; deWaard et al. 2019; Lopez‐Vaamonde et al. 2021). The most used universal primer set for barcoding invertebrates using CO1 (LCO1490/HCO2198; Folmer et al. 1994) does not overlap with the region initially used by Danforth et al. (1998) to separate *H. ligatus* and *H. poeyi* (C1‐J‐2183/TL2‐N‐3014; Simon et al. 1994). Further, we found that these universal primers were not specific enough for developing a comprehensive database of *Halictus* samples from NC that could be combined with existing datasets to define the geographic distribution of these two species. In response, we developed new primers to amplify a region of *Halictus* CO1 that can be used to distinguish between *H. ligatus* and *H. poeyi* across the mid‐Atlantic region of the Eastern USA, while allowing for integration with public datasets.

The *Halictus* species complex highlights the importance of properly identifying cryptic species. Bees, in many terrestrial biomes, provide the critical ecosystem service of pollination to the vast majority of flowering plants and many of the world's most important crops (Ollerton et al. 2011; Klein et al. 2006; Khalifa et al. 2021). A variety of environmental stressors (Vanbergen et al. 2013) currently threaten pollinator populations, with some taxa experiencing documented declines (Cameron et al. 2011). Many bee species are already difficult to properly identify using morphology alone, and the presence of cryptic species only further complicates this issue, limiting conservation efforts. Considering these two *Halictus* species as but one example demonstrates the benefits of reliable molecular tools; by defining a more accurate range boundary, as well as identifying zones of overlap, issues such as resource use, environmental tolerances, possible competitive interactions, and evolutionary divergence can be more thoroughly addressed. With increasing concerns about native bee conservation, as well as recent efforts to develop improved monitoring and conservation planning (Woodard et al. 2020), defining the distribution of cryptic species using reliable molecular tools will provide critical information for these efforts.

Given these research needs, our goals were to (1) develop a robust, inexpensive molecular identification protocol for *H. ligatus* and *H. poeyi* , (2) examine genetic structure and phylogeographic patterns as related to previous hypotheses regarding lineage divergence, and (3) improve the species range delineations using a combination of sequences generated in this study and publicly available data.

## 2 Materials and Methods

### 2.1 Physical Sample Collection and DNA Extraction

**FIGURE 1:**
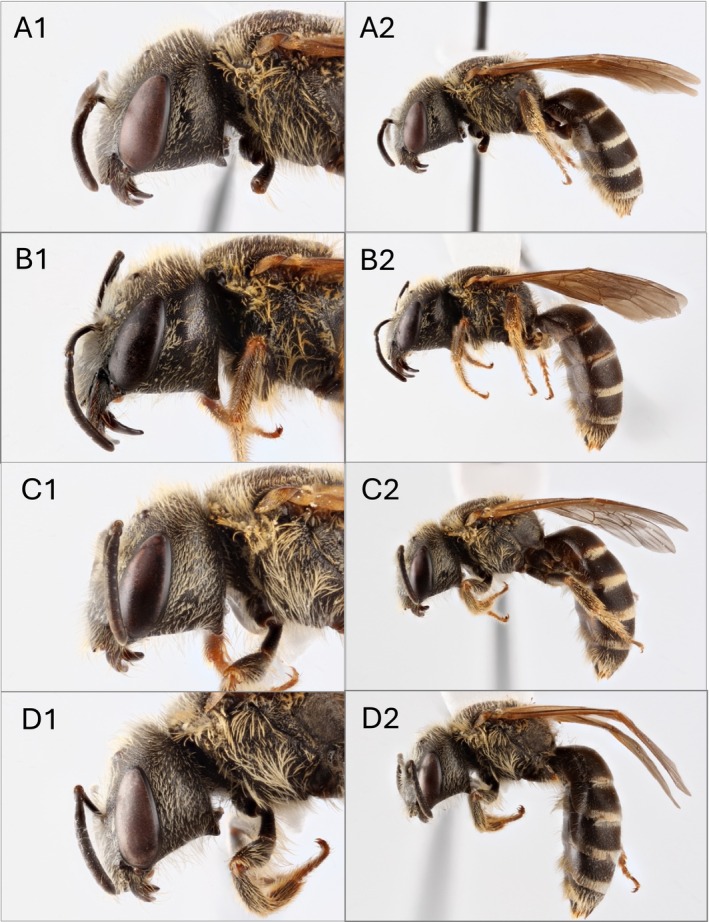
Confirmed specimens of *Halictus ligatus* and *H. poeyi* using the HAL primers, with the left column showing a lateral view of the head and the right column showing a lateral view of the entire specimen. Photos (A1) and (A2) are *H. poeyi* from collection site CI; photos (B1) and (B2) are *H. poeyi* from collection site PM (where both *H. poeyi* and *H. ligatus* are present), (C1) and (C2) are *H. ligatus* from collection site PM; and (D1) and (D2) are *H. ligatus* from collection site MN. Legs from these specimens were taken for molecular testing. Photograph credit: Matt Bertone at NC State University.

Physical specimens of *Halictus ligatus* and *H. poeyi* were collected in North Carolina (NC), USA, in 2017 from 12 of the NC Department of Agriculture & Consumer Services' Experimental Agricultural Research Stations (Table 1; Figure 1). Briefly, samples were collected as part of a larger study (see Levenson and Tarpy 2023 for more details) by sweep net between April and September. Samples were transported from the field to the lab on ice and stored at −20°C until further processing. Specimens of this species complex were initially morphologically identified as *Halictus ligatus/poeyi*, with no further morphological identifications conducted.

**TABLE 1:** Research Station locations where samples were collected with *Halictus ligatus* found at 5 locations and *H. poeyi* found at 10 locations.

Sample location	Sample size	*H. ligatus*	*H. poeyi*
Border Belt Research Station (BB)	7*		✓
Central Crops Research Station (CC)	8		✓
Horticultural Crops Research Station, Clinton (CI)	8		✓
Caswell Research Station (CW)	8		✓
Lake Wheeler Research Station (LW)	8	✓	✓
Mountain Research Station (MN)	8	✓	
Mountain Horticulture Research Farm & Extension Center (MH)	8	✓	
Oxford Research Station (OX)	8	✓	✓
Peanut Belt Research Station (PB)	8		✓
Piedmont Research Station (PM)	8	✓	✓
Sandhills Research Station (SH)	8		✓
Upper Piedmont Research Station (UP)	8		✓

*Note:* Three locations had both species. See Levenson and Tarpy 2023 for more details on specimens.

* Two of these samples were *Lasioglossum lustrans* to act as an outgroup.

In 2018, DNA was extracted from female specimen legs using Hotshot (Truett 2000) in the NC State Apiculture Program's laboratory at NC State University (NCSU). DNA was extracted from a total of 96 samples: 8 samples from each of the 12 sampling locations except for one (Border Belt Tobacco Research Station), where DNA was extracted from 7 samples and an additional positive *H. poeyi* sample from Florida that was collected in 2012. Two of the samples included from Border Belt Tobacco Research Station were *Lasioglossum lustrans* , which were utilized as outgroup taxa where applicable in phylogenetic analyses. DNA concentration and purity were measured using a NanoDrop 2000 v 1.6 spectrophotometer (Thermo Scientific, Wilmington, Delaware, USA). Extracted DNA was then stored at −20°C until it was shipped overnight on ice to Colorado State University (CSU) for molecular analysis. To address issues in Sanger sequencing (see below), new legs from 42 of the initial 96 specimens were removed in 2023 and shipped overnight on ice to CSU for re‐extraction. New extractions were conducted using the Qiagen DNeasy Blood and Tissue Kit (Qiagen Inc., Hilden, Germany) following the manufacturer's protocol. The DNA was quantified by both NanoDrop and Qubit (Thermo Fisher Scientific, Waltham, Massachusetts, USA).

### 2.2 PCR, Sequencing, and Primer Development

Once DNA was successfully extracted from all samples, PCR was carried out using a Bio‐Rad C1000 Touch thermal cycler (Bio‐Rad Laboratories Inc., Hercules, California, USA). Initially, universal CO1 primers (LCO1490 and HCO2198; Folmer et al. 1994)—hereafter referred to as universal primers—were used to generate sequences that could be aligned to public *Halictus* CO1 datasets. The 50 μL reactions contained 37.75 μL molecular grade H_2_O, 5.00 μL 10× Ex Taq buffer, 4.00 μL dNTP mixture at 2.5 mM, 200 nM LCO1490 forward primer, 200 nM HCO2198 reverse primer, 1.25 U of TaKaRa Ex Taq HS polymerase (Takara Mio Inc., Shiga, Japan), and 1 μL of DNA template of varying concentration. The thermocycler protocol used on all PCRs presented here included an initial denaturation step of 94°C for 3 min, followed by 40 cycles of 94°C for 20 s, 50°C for 20 s, 72°C for 30 s, and a single final extension step at 72°C for 5 min. A lid temperature of 105°C was maintained throughout all cycles. After PCR, amplicons were run on a 1% agarose gel to evaluate success. A subset of 16 samples that did not initially produce bands on the agarose gel with the universal primers were re‐run with the same primers and a PCR protocol that contained 10% more TaKaRa Ex Taq HS polymerase, 10× Ex Taq buffer, and dNTP mixture and proportionally less water to make up a total reaction volume of 50 μL. A total of 91 PCR products were purified with a Qiagen QIAquick PCR purification kit following the manufacturer's protocol (Qiagen Inc., Hilden, Germany). Purified amplicons were sent to the University of Chicago Comprehensive Cancer Center DNA Sequencing Facility for Sanger sequencing using the same primers as for amplification on an Applied Biosystems 3730XL DNA sequencer (Applied Biosystems, Foster City, California, USA). Samples that produced faint bands on the agarose gel were re‐run with the same PCR protocol with the addition of 2.5 μL of MgCl_2_ at 25 mM and 100 ng of BSA to improve amplification. All successful PCR products from the DNA re‐extractions were purified as described above and sent to Azenta (Azenta US Inc., South Plainfield, New Jersey, USA) for Sanger sequencing on an Applied Biosystems 3730XL.

In response to the low sequencing success rate, we developed new, *Halictus*‐specific CO1 primers (HAL_134_F and HAL_958_R; hereafter referred to as HAL primers; Table 2). The design of the new primers employed a DNA alignment (see methods below) of our previously generated DNA sequences, publicly available CO1 sequences, and whole mitogenome data from *Halictus*. From the alignment, regions of maximum similarity were chosen for automated primer design using Primer3 v. 0.4.0 (Untergasser et al. 2012) using the following settings: divalent cations = 1.5; monovalent cations = 50; dNTPs = 0.6 mM; and the Santa Lucia (1998) formula for thermodynamic parameters and salt correction. To test these new HAL primers, 22 samples that did not produce sequences under the universal primers were again subjected to PCR with the same standard reaction conditions (without additives) using 400 nM HAL primers and less water to make up the 50 μL reaction. All PCRs, except the positive *H. poeyi* , produced a strong band on 1% agarose gels. Amplicons were purified with the Qiagen QIAquick PCR purification kit following the manufacturer's protocol (Qiagen Inc.) and sent to Azenta for Sanger sequencing using the HAL primers as described above. We then compared the results with those from the universal primers (Appendix S1).

**TABLE 2:** New *Halictus*‐specific CO1 primers (HAL primers), HAL_134_F and HAL_958_R.

Primer name	Locus	Primer sequence	*T*_*m*_ (°C)
HAL_134_F	CO1	GGAAGATGAATTAATAAYGATCAAA	55.9–57.6
HAL_958_R	CO1	CTTTAATTCCTGTTGGTACA	52.3

### 2.3 Sequence Analyses, Species Tests, and Phylogeography

Geneious Prime (v. 2024.0.5; www.geneious.com) was used to manually trim the poor‐quality base calls and primer sequences from the 5′ and 3′ ends, assemble forward and reverse sequences into contigs, and generate consensus sequences from the contigs. The consensus sequences were aligned in Geneious using MAFFT v. 7.490 (Katoh et al. 2002; Katoh and Standley 2013) and trimmed to a consistent length (566 bp). For some analyses, additional CO1 sequences from the closely related species *Halictus rubicundus* were downloaded from GenBank (Appendix S2) as an outgroup taxon. The generated and downloaded sequences were aligned and trimmed to a consistent length (543 bp). Bayesian trees were generated from each version of the alignment using MrBayes v. 3.2.6 (Huelsenbeck and Ronquist 2001; Ronquist and Huelsenbeck 2003) with a GTR substitution model, MCMC chain length of 1,100,000, subsampling frequency of 200, and a 100,000 tree burn‐in with a molecular clock and a uniform branch length prior using a Gamma of 1,1. *Lasioglossum lustrans* was set as the outgroup in each case.

Haplotypes were determined from aligned sequences trimmed of lower‐quality ends and were called manually from the 566 bp alignment. Genetic diversity metrics for *H. poeyi* and *H. ligatus* were calculated using DnaSP v6.12.03 ×64 (Rozas et al. 2017). Calculations included the number of haplotypes (h), number of polymorphic (segregating) sites (S), haplotype diversity (Hd), nucleotide diversity (*π*), and average number of nucleotide differences (k) within each species (Table 3). An additional Bayesian tree was generated with a subset of individuals representing each haplotype from each collection location using the parameters described above and including the *H. rubicundus* and *L. lustrans* outgroups. This tree was used to test for cryptic species using the Species Delimitation plugin (Masters et al. 2011) for Geneious Prime. Each monophyletic internal clade that resolved within *H. poeyi* and *H. ligatus* was compared to their most closely genetically related clade to assess current species boundaries. The sample set was reduced to avoid a calculation artifact that exists within the Species Delimitation program (see Masters et al. 2011).

**TABLE 3:** Comparison of molecular diversity metrics between *H. poeyi* and *H. ligatus* from the generated 566 bp CO1 gene fragment.

	*n*	Number of haplotypes (h)	Number of polymorphic (segregating) sites (S)	Haplotype diversity (Hd)	Nucleotide diversity (*π*)	Average number of nucleotide differences (*k*)
*H. poeyi*	66	5	6	0.675	0.00330	1.866
*H. ligatus*	21	7	12	0.829	0.00481	2.724

To determine phylogenetic and geographic differentiation within and between *H. poeyi* and *H. ligatus* in NC, a TCS statistical parsimony analysis (Clement et al. 2002) was run in PopART v. 1.7 (Population Analysis with Reticulate Trees; Leigh and Bryant 2015) to create a haplotype network. An additional modified Bayesian tree was created following the above parameters from a reduced number of sequences with one individual of each haplotype from each collection location and only *L. lustrans* as the outgroup. The tree was mapped geographically onto each of the collection locations using GenGIS v. 2.5.3 (Parks et al. 2013) to determine if there were geographically isolated branching patterns within either species.

The R‐based program (R Core Team 2023) Geneland v. 4.0.0 (Guillot, Estoup, et al. 2005; Guillot, Mortier, et al. 2005; Guillot et al. 2008; Guillot 2008; Guillot and Santos 2010; Guedj and Guillot 2011) was employed to test hypotheses about the influence of soil characteristics on the geographic range of *H. ligatus* and *H. poeyi* in NC. Several different Markov Chain Monte Carlo simulations in Geneland were run in order to explore what factors may have historically affected current species ranges and haplotypic divergence. The simulations included haplotype data only, haplotype with geospatial coordinates, haplotype with average soil moisture, haplotype with average soil temperature, and haplotype with estimated geologic age of parent material (a proxy for soil type/physical characteristics). Parameters for the Geneland runs were as follows: 20 independent runs (an additional 20 were run if multiple nearly equal partitions were resolved), 100,000 iterations per run, every 100th iteration held in memory, 200 postprocessing burn‐in, uncorrelated allele frequencies, and a value of 360 for uncertainty on coordinates when using the spatial model to allow for migration between populations and so that individuals from the same location did not bias the spatial model. Individual bar plots and spatial tessellation were employed to visualize the Geneland results. To visualize patterns in landscape values, collection site longitude (*x* axis) was regressed on average soil moisture, average soil temperature, and geologic age of parent material, separately, and the *R* ^2^ calculated. The soil data were retrieved from Web Soil Survey (Soil Survey Staff, n.d.) using a 0.5 km radius at each sampling location using ArcGIS Pro (version 3.1.0, Environmental Systems Research Institute, Redlands, California, USA).

To put our NC sequences in the context of broader geographic *Halictus* sampling, additional CO1 sequences for *H. ligatus* and *H. poeyi* as well as sequences for *Halictus townsendi* (another cryptic species (Packer et al. 2016)) and sequences for a collection of *Halictus* sp. specimens from Bentsen‐Rio Grande Valley State Park in Texas were downloaded from the Barcode of Life Data System (Bold Systems; Ratnasingham and Hebert 2007; Ratnasingham and Hebert 2013; Ratnasingham 2024). All available data were downloaded and filtered for sequences with more than 400 bp overlapping with our newly generated sequences. After filtering the available datasets, we used 15 additional sequences of *H. poeyi* , 510 additional sequences of *H. ligatus* , 12 sequences from *H. townsendi* , and 6 *Halictus* sp. sequences from Texas. All sequences were aligned using MAFFT with the parameters outlined above. The final alignment was 439 bp long and consisted of 630 *Halictus* sequences. This data matrix was used to repeat the TCS Network to visualize haplotype diversity following the parameters outlined above with sequences characterized by collection state or province and country. The alignment was repeated, including *L. lustrans* as an outgroup, and a neighbor‐joining tree was generated using the Tamura‐Nei (Tamura and Nei 1993) genetic distance model and jackknife resampling of 1000 replicates along with a Bayesian tree following the parameters outlined above.

## 3 Results

The commonly used universal CO1 primers LCO1490 and HCO2198 (Folmer et al. 1994) were initially used to barcode *Halictus* specimens for this project, but due to problems associated with non‐specific binding, poor‐quality Sanger sequences were returned. To remedy this issue, we designed new CO1 primers specific to *Halictus* (HAL primers). The new primers increased the specificity and quality of Sanger sequences (Appendix S1). Mitogenome sequence alignments used to design new primers confirmed the mismatches in the previously employed primer set. Amplicons generated from the redesigned primers overlap sufficiently with publicly available CO1 sequences such that these new primers can be used for future *Halictus* identification projects.

**FIGURE 2:**
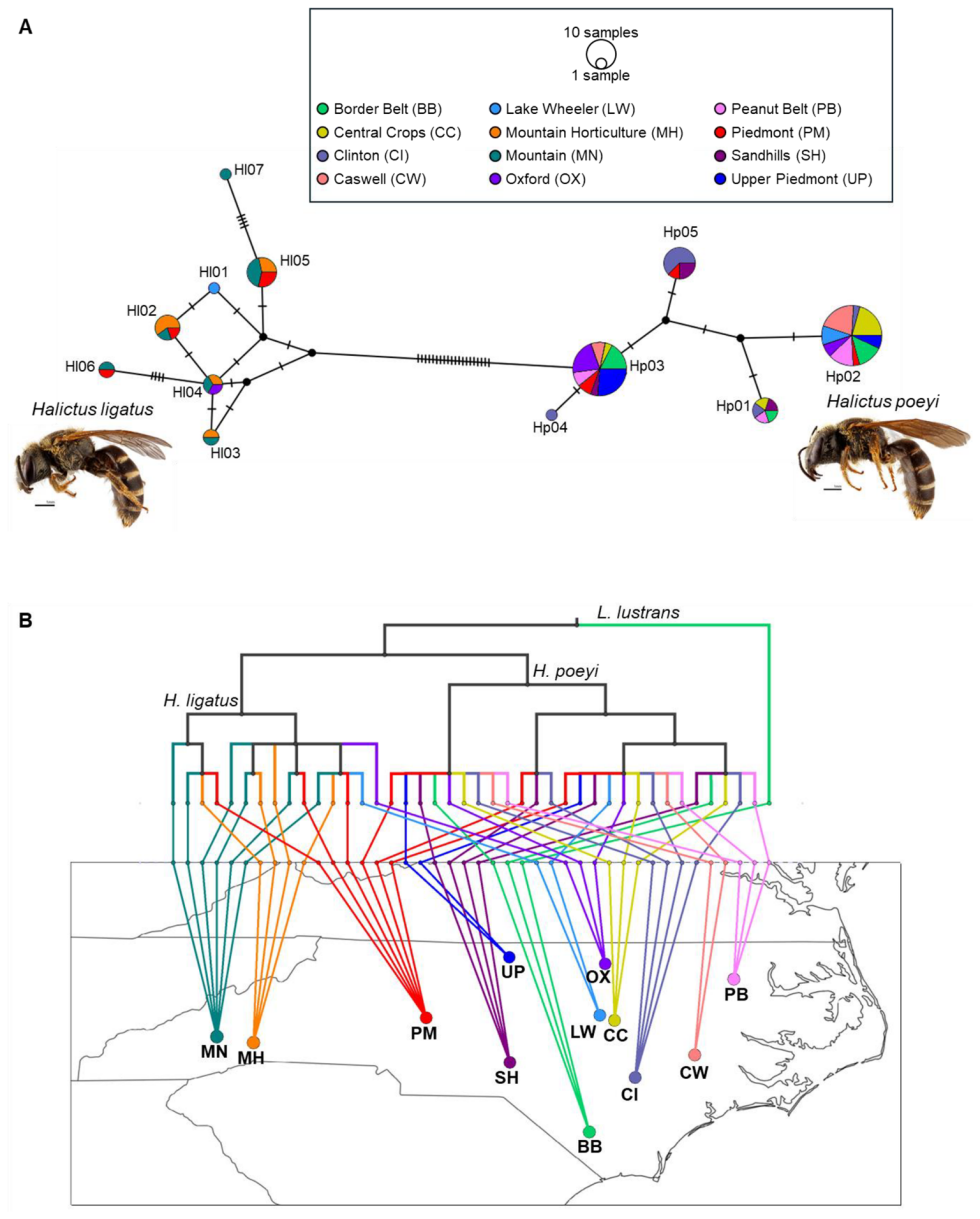
Haplotype and location data for each specimen of *Halictus* collected for this study reveal no spatial differentiation between individuals for either species. (A) A TCS network of haplotype diversity for *H. ligatus* (left) and *H. poeyi* (right) from CO1 sequence data. Informative SNVs separating the two species and each haplotype are indicated by hatchmarks. Nodes indicate likely uncollected haplotypes. Each haplotype is proportional to its sample size and is divided into a pie chart indicating the number of individuals collected from each location. Images are the same specimens as depicted in (B2) and (C2) in Figure 1. Photograph credit: Matt Bertone at NC State University. (B) A Baysian tree showing relationships between each haplotype from each collection location is mapped geographically. All specimens of *H. ligatus* resolved to the left branch of the tree, and all specimens of *H. poeyi* resolved to the right branch of the tree. The tree is rooted using *Lasioglossum lustrans* (collected from BB). Each terminal is colored to correspond to a collection location. Collection locations are labeled with a two‐letter code and color coded corresponding with the key in subfigure (A).

With the new HAL primers, we were also able to successfully barcode 87 specimens (Genbank accessions PQ576549‐PQ576636) from NC. In a Bayesian analysis of the generated CO1 sequence data, *Halictus ligatus* and *H. poeyi* collected in NC segregate into two well‐supported clades (Appendix S2). Within *H. ligatus* , we sampled 21 individuals that were sorted into seven haplotypes from five locations (Lake Wheeler Research Station (LW), Mountain Research Station (MN), Mountain Horticulture Research Farm & Extension Center (MH), Piedmont Research Station (PM), and Oxford Research Station (OX); Figure 2). A total of 66 *H. poeyi* were sampled, which were sorted into five haplotypes from 10 locations (Border Belt Tobacco Research Station (BB), Caswell Research Station (CW), Central Crops Research Station (CC), Horticultural Crops Research Station, Clinton (CI), LW, OX, Peanut Belt Research Station (PB), PM, Sandhills Research Station (SH), and Upper Piedmont Research Station (UP); Figure 2).

Haplotype diversity within NC was visualized using a TCS network, which showed a lack of geographic specificity of each haplotype for both species with the exception of Hl01 and Hl07, which were each sampled only once (Figure 2A). Greater haplotype and molecular diversity were present in *H. ligatus* than in *H. poeyi* despite the smaller geographic (in this study) and specimen sample sizes (Table 2A). The east–west geographic divide between *H. poeyi* and *H. ligatus* is represented on the GenGIS plot of the Bayesian tree mapped onto the NC sample sites (Figure 2B). The majority of *H. poeyi* specimens fell within two haplotypes (Hp02, *n* = 29, or Hp03, *n* = 23). Specimens of *H. ligatus* were more evenly spread across seven haplotypes with Hl05 being the most commonly sampled (*n* = 7). Both species show broad geographic distribution of all haplotypes with no segregation of individual haplotypes at a single geographic location. There is geographic partitioning between the two species, with *H. ligatus* contained to the western half of the state with some populations in the montane central region (MN and MH had *H. ligatus* only) and *H. poeyi* present in the lower elevation, eastern half of the state (BB, CW, CC, CI, PB, SH, and UP had *H. poeyi* only). The two species' ranges overlap at three collection locations in the central, piedmont region of the state (LW, OX, and PM).

**FIGURE 3:**
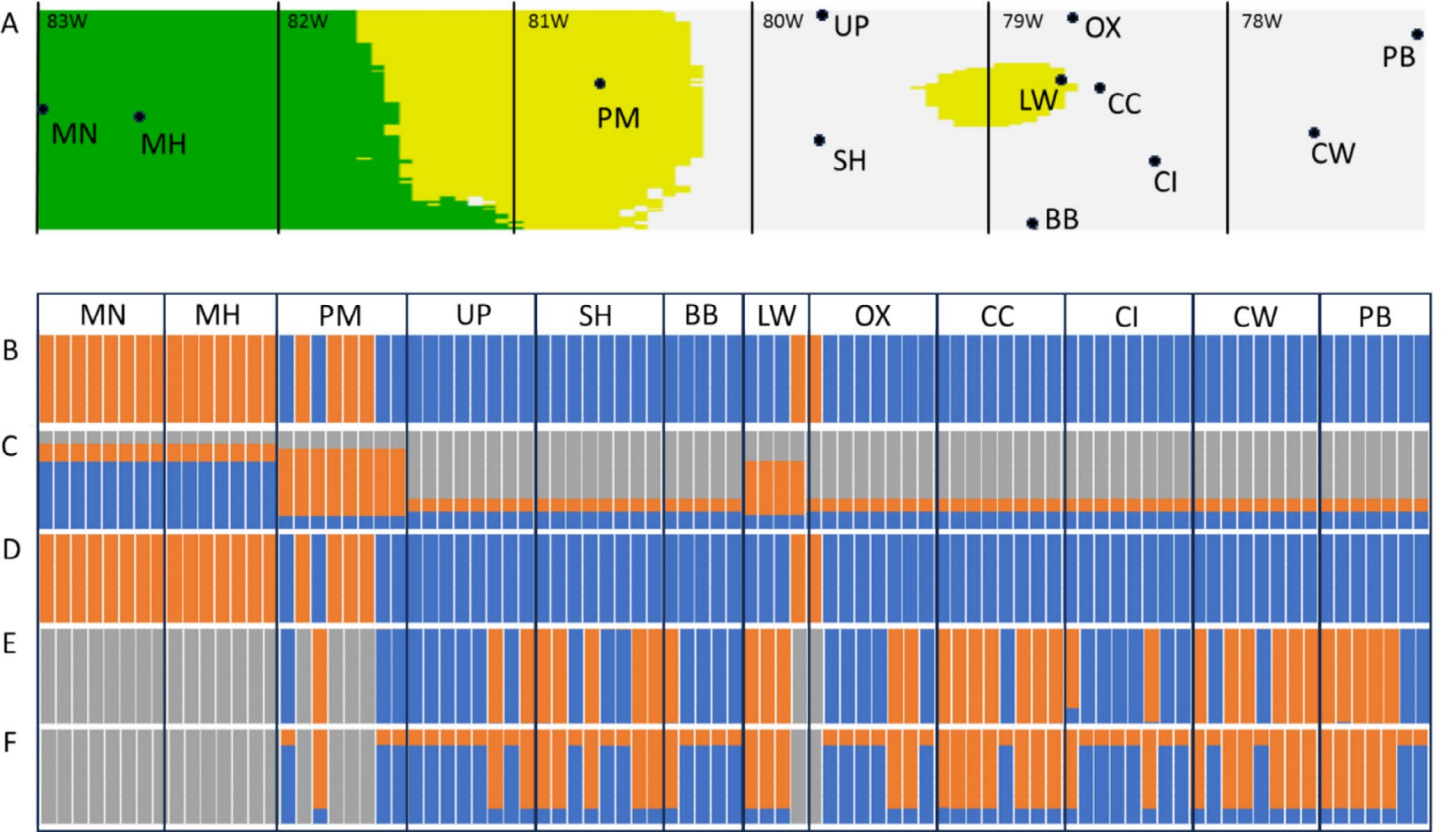
Population structure inferred from haplotypic data and combined with different geographic variables. (A) A geographic population structure map inferred from haplotypic and geographic data (see barplot C), where green represents predicted regions with *H. ligatus* only, yellow regions of *H. ligatus* and *H. poeyi* co‐occurrence, and white regions with *H. poeyi* only. Points with two‐letter abbreviations are collection sites (refer to Figure 2 and Table 1 to cross‐reference location abbreviations), and vertical lines indicate longitude. (B) A barplot of individuals (each bar represents an individual) with percent membership (amount of each color in each bar) inferred from haplotypic data only (membership is orange for *H. ligatus* and blue for *H. poeyi* ). (C) Population structure inferred from haplotypic data and geographic location (membership is blue for *H. ligatus* , orange for evenly sympatric sites, and gray for *H. poeyi* ). (D) Population structure inferred from haplotypic data and soil moisture at collection sites (membership is orange for *H. ligatus* and blue for *H. poeyi* ). (E) Population structure inferred from haplotypic data and soil temperature at collection sites (membership is gray for *H. ligatus* , blue for *H. poeyi* subpopulation containing haplotypes Hp03, Hp04, and Hp05, and orange for an *H. poeyi* subpopulation with 91% of individuals either possessing haplotypes Hp01 or Hp02). (F) Population structure inferred from haplotypic data and the geological age of soil parent material at collection sites (membership is gray for *H. ligatus* , blue for an *H. poeyi* subpopulation containing haplotypes Hp03, Hp04, and Hp05, and orange for a *H. poeyi* subpopulation with 91% of individuals either possessing haplotypes Hp01 or Hp02). The order of samples is the same across all barplots. See Appendix S3 to view soil property metrics regressed on geographic locations.

The patterns of genetic structure resolved from the Geneland analyses (Figure 3) suggest that nucleotide differences between CO1 sequences (i.e., between the two different species) are an important factor in the geographic patterns seen in the Geneland outputs even when soil property parameters were added to the analysis. As such, correspondences among geographic locations, soil characteristics, and species haplotypes were observed (Figure 3; Appendix S3), with *H. ligatus* noted from western sites that possessed soils that were generally wetter, cooler, and from geologically older parent material. The results from the combined soil moisture and haplotype analysis separated individuals by species (*k* = 2) designations, with an average water fraction by volume for the *H. ligatus* sites being 0.36 and water fraction by volume being 0.26 on average for the *H. poeyi* sites (Figure 3D). In the analyses where soil temperature and geologic age of parent material were run with haplotype data, the most optimal population partitions in both cases were *k* = 3 (Figure 3E,F). The average soil temperature for the *H. ligatus* sites was 16.28°C; 17.67°C for the *H. poeyi* subpopulation (blue bars in Figure 3E) made up of individuals with haplotypes Hp03 (*n* = 23), Hp04 (*n* = 1), or Hp05 (*n* = 7); and 17.54°C for the *H. poeyi* subpopulation (orange bars in Figure 3E) made up of individuals with haplotypes Hp01 (*n* = 4), Hp02 (*n* = 28), Hp03 (*n* = 1), or Hp05 (*n* = 2). The average geologic age of the parent material underlying the *H. ligatus* sites was 740.4 mybp (million years before present); 269.8 mybp for the *H. poeyi* subpopulation (blue bars in Figure 3F) made up of individuals with haplotypes Hp03 (*n* = 23), Hp04 (*n* = 1), or Hp05 (*n* = 7); and 213.5 mybp for the *H. poeyi* subpopulation (orange bars in Figure 3F) made up of individuals with haplotypes Hp01 (*n* = 4), Hp02 (*n* = 28), Hp03 (*n* = 1), or Hp05 (*n* = 2). The *H. poeyi* subpopulation (blue bars in Figure 3E,F) made up of individuals with haplotypes Hp03 (*n* = 23), Hp04 (*n* = 1), or Hp05 (*n* = 7), had a more western geographic distribution based on average longitude of individuals across sites. The haplotypes Hp03, Hp04, and Hp05 resolve as more early diverging than Hp01 and Hp02 in our phylogenetic analyses (Appendix S5A,B).

To examine patterns of selection within and between *H. ligatus* and *H. poeyi* in NC, each species clade and the subclades within them were tested for the presence of cryptic speciation (Appendix S4). Low intra/interspecies variation and statistically significant Rosenberg's P(AB) indicate that the differences among clades within each species are small relative to the reciprocally monophyletic closest species. Furthermore, each branching event corresponds to the expected random coalescent model of a Wright‐Fisher population.

**FIGURE 4:**
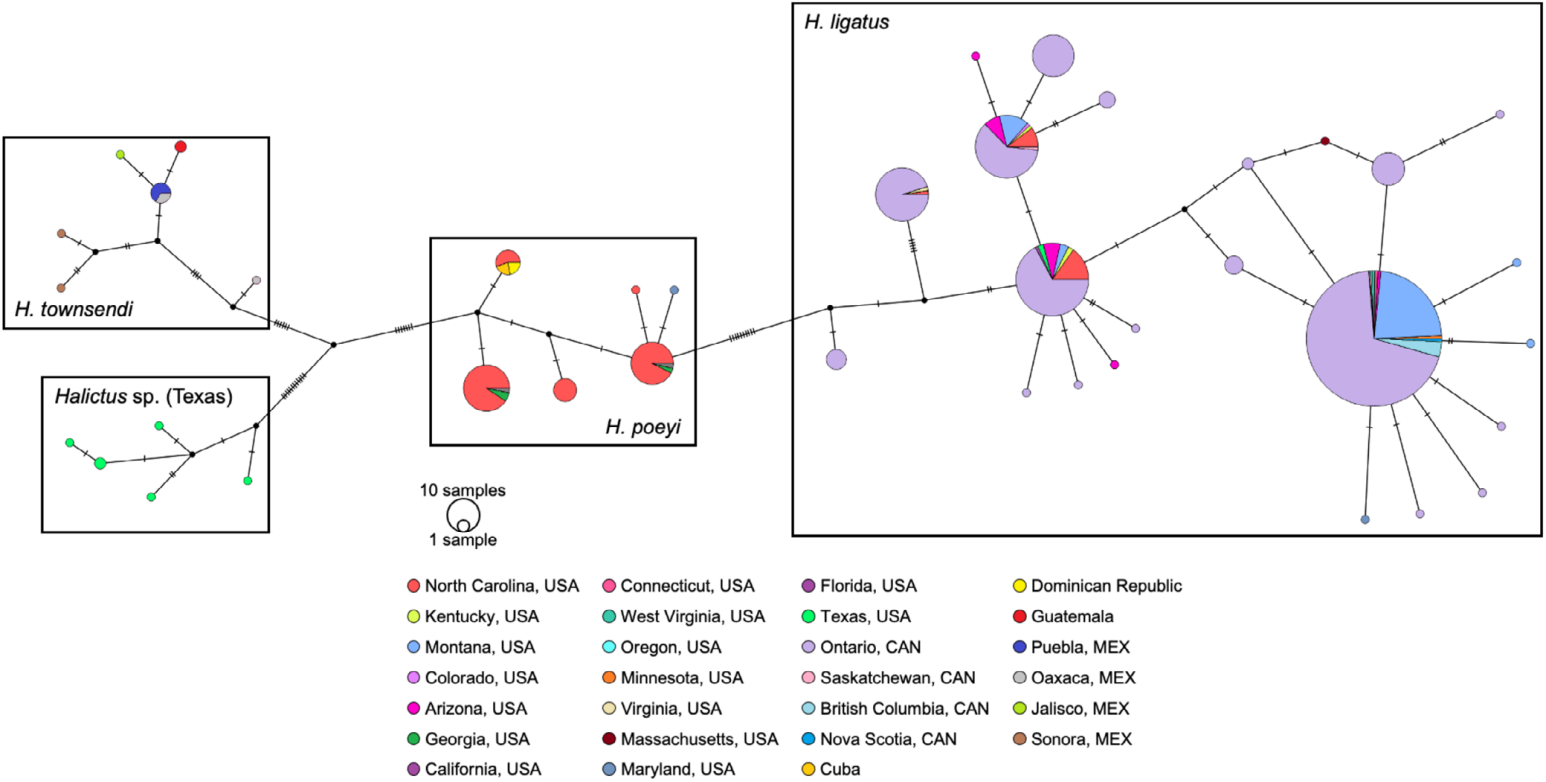
TCS network of haplotype diversity by location for *H. ligatus* , *H. poeyi* , *H. townsendi* , and an unnamed cryptic species in Texas previously thought to be *H. ligatus* (*Halictus* sp. Texas). All sequences except those from NC, USA, were aggregated from BOLD systems. Informative SNVs separating each species and within‐species haplotypes are indicated by hatchmarks; nodes indicate likely uncollected or extinct haplotypes. Each haplotype is shown as a pie chart indicating the proportion of individuals in that haplotype by collection location. The size of the circle representing each haplotype is proportional to the total number of individuals collected in each haplotype, which ranges from 1 to 274.

A subset of publicly available barcode data was combined with our sequences to put our results in the context of more broadly sampled *Halictus* species CO1 sequences and to more comprehensively analyze the geographic range of each species (Appendices S5 and S6; also see Appendix S7). The TCS network was repeated with the new samples categorized by collection location (Figure 4). After the addition of public data, specimens of *H. poeyi* ranged from the Dominican Republic at the southernmost point to Maryland, USA, at the northernmost point, although most were concentrated in the Southeastern USA. In this larger dataset, the samples from NC encompassed all but one of the haplotypes found in the dataset for *H. poeyi* . Specimens from *H. ligatus* were sampled from Arizona, USA, at the southernmost extent, to Southern Canada at the northernmost extent, and from both the East and West Coasts of the USA. With the truncated alignment at 439 bp, our NC *H. ligatus* sequences sorted into four haplotypes, all of which were collected elsewhere in the range. The combined dataset of *H. ligatus* in this alignment was sorted into 23 total haplotypes. There were no records of either *Halictus* species in NC available in the public data in BOLD Systems. Sequences from two putative cryptic *Halictus* species each resolved in distinct haplotype groupings in the TCS network as they are labeled in BOLD: *Halictus* sp. and *H. townsendi* (Figure 4).

## 4 Discussion

The distributions of the two cryptic species *Halictus ligatus* and *H. poeyi* across North Carolina (NC), USA, loosely follow the accepted distributions of the two species across the Eastern USA seaboard; *H. ligatus* is present in more northern or higher elevation locations (Mountain Research Station and Mountain Horticulture Research Farm & Extension Center), while *H. poeyi* is present in more southern or lower elevation locations (Border Belt Tobacco Research Station, Caswell Research Station, Central Crops Research Station, Horticultural Crops Research Station at Clinton, Peanut Belt Research Station, Sandhills Research Station, and Upper Piedmont Research Station). There are three locations in NC, all in the Piedmont region of the state, where both species are present (Lake Wheeler Research Station, Oxford Research Station, and Piedmont Research Station). The two species are distinct from each other, and there appears to be no further speciation within either group in NC based on geographic or haplotypic segregation (Appendix S4; Figures 2 and 4). It has been suggested that the altitudinal gradient (east to west) across NC recapitulates the latitudinal gradient from Florida to Maine in the USA (Mitchell 1960). Our findings from these two *Halictus* species support that assertion, making NC an excellent natural laboratory for this species complex as well as other pollinator species.

Our newly developed primers represent an improvement over the use of universal barcoding primers in that they are more specific to *Halictus* CO1 sequences and, as a result, produce fewer off‐target amplifications and higher quality base calls throughout the sequence (Appendix S1). The universal primers used by Danforth et al. (1998) are part of a large set of universal primers developed by Simon et al. (1994) for the amplification of various regions of mitochondrial DNA. The forward primer used by Danforth et al. (1998) (C1‐J‐2183) binds to the 3′ region of CO1, a region of the gene that is not used as often for barcoding, and the reverse primer (TL2‐N‐3014) binds to a tRNA coding region outside of CO1 that includes a variably sized indel region across invertebrate taxa (see Simon et al. 1994). The universal primers (LCO1490 and HCO2198) we first used to amplify *Halictus* CO1 for this study bind to the 5′ region of CO1, which is colloquially known as the “Folmer region” and is widely employed in genetic studies due to the high frequency of SNVs in this region (Folmer et al. 1994). Other universal CO1 primers have been developed for bees more recently but rely on high‐throughput parallel sequencing and read filtering to attain high quality sequences (Creedy et al. 2020) or permissive thermocycling conditions to attain sequences (Françoso and Arias 2013). Both of these approaches were inapplicable to our *Halictus* study due to scale, cost, and/or resulting sequence quality and were therefore not employed. The locus amplified by our newly designed primers overlaps with the Folmer region for approximately 400 bp, enabling us to combine our data with publicly available sequences (Figure 4). Based on the sequence diversity we detected, additional sampling will be needed to fully capture all haplotypes of these two species where they have overlapping species ranges and in under sampled regions.

Using our data and public barcode data, we were able to interrogate the region within the USA where *H. ligatus* and *H. poeyi* co‐occur. Using the aggregated data, it appears that this region exists in a line that runs along the foothills of the Appalachian Mountains from at least Anne Arundel County, Maryland, USA, at the northernmost extent to Atlanta, Georgia, USA, in the south (Appendix S6). Both species have been reported at 8 total collection locations, including the 3 reported here, in the USA, all but one of which (Chattanooga, Tennessee, USA; see Carman and Packer 1996) have available CO1 sequence data. Carman and Packer (1996) first found evidence of *H. poeyi* using allozyme data and stated the border of the two species' ranges was the southern margin of the Appalachian Mountains. Collections of *H. poeyi* north of this area since then suggest otherwise. When Carman and Packer (1996) first discovered *H. poeyi* in Florida, they were investigating populations of what was thought to be *H. ligatus* that had differing life histories in which some populations lived in persistent year‐round nests, while others were present only seasonally. Both populations proved to be *H. poeyi* and did not differ molecularly from each other (Carman and Packer 1996). There are no clear behavioral differences separating *H. poeyi* and *H. ligatus* . Furthermore, because *H. poeyi* is known to exhibit multiple nesting behaviors in Florida, independent of genetic differentiation (Zayed and Packer 2002), we must also consider whether this factor may affect seasonal variation across the *H. poeyi* species range. Additional sampling and genetic characterization of these populations from a broader geographic region and from longitudinal temporal sampling will help clarify the observed genetic diversity in *H. poeyi* . Additionally, observing the arrival time of each species at foraging locations where both have been collected, for example, at Oxford, Lake Wheeler, and Piedmont Research Stations in NC, may clarify whether interspecies competition for food or nesting resources is occurring between these two species. It has previously been observed that when *H. ligatus* and *H. poeyi* are in sympatry, they are more similar to each other than allopatric populations of each species (Dunn et al. 1998), indicating that the differences between species are more likely due to environmental or micro‐climatic differences between nest locations.

To further examine differences in species range between *H. ligatus* and *H. poeyi* in NC, we utilized Bayesian methods (i.e., Geneland) to analyze haplotypic diversity in combination with geographic locations and soil properties. The results from the Geneland runs support the results produced from other analyses (TCS networks and Bayesian tree) that employed haplotype sequence data (Figures 2 and 4). We chose to examine soil temperature, moisture, and geologic age of parent material given that they are known to be essential environmental components in the ecological adaptation of the vast majority of wild bees—most bee species, including *Halictus*, nest underground—and yet little quantitative work has been conducted at large scales to understand the interplay between bee species and soil characteristics (Antoine and Forrest 2021). Of the three soil properties, only moisture resolved clusters corresponding with *H. ligatus* and *H. poeyi* haplotypes, whereas both soil temperature and geologic age of parent material were split by species, and then within *H. poeyi* into two subpopulations: one associated with haplotypes Hp03, Hp04, and Hp05 and a second with Hp01 and Hp02 (Figure 3). Of the three soil characteristics, the geologic age of parent material was the most obviously different between the subpopulations (Appendix S3). This finding is perhaps unsurprising given that the age of parent material (also a proxy for type of parent material in the case of NC with older granitic rocks in the west and younger sedimentary deposits in the east) is a strong determining factor in soil formation (e.g., Jenny 1941; Raab et al. 2017). Soil texture and composition are thought to be important criteria for nest site selection among ground‐nesting bees (e.g., Cane 1991; Potts and Willmer 1997; Leonard and Harmon‐Threatt 2019). While soil temperature and moisture differed less across NC, they could still be found to correspond with differences in haplotypes across the landscape and are also known to be important criteria for nest selection among ground‐dwelling bees (e.g., Packer and Knerer 1986; Stone 1994; Wuellner 1999; Weissel et al. 2006). In all, the results from these soil by haplotype analyses suggest that these methods should be expanded to other parts of the species ranges' to better understand the role of soil type in determining species occupancy. Our results here also present a series of testable hypotheses, such as *H. ligatus* individuals prefer nesting sites in soil from older/granitic parent material. Interestingly, most of Ontario, the source of most available *H. ligatus* sequence data (see Figure 4), resides on parent material that is of Precambrian age, and much of that is Archean (Ontario Geological Survey 2019).

Given the genetic diversity we found for these two *Halictus* species in NC, a larger‐scale comparison from across the species ranges was warranted to better assess patterns observed in our dataset. Within NC, using a 566 bp fragment of CO1, we found five haplotypes within *H. poeyi* and seven haplotypes within *H. ligatus* (Figure 2). Specimens of *H. poeyi* were largely sorted into two main haplotypes (Hp02 and Hp03, Figure 2), while *H. ligatus* individuals were more evenly spread across haplotypes. This pattern extends to the larger comparative dataset (Figure 4). For the full dataset, we trimmed the alignment to 439 bp to avoid possible issues with long branch attraction (Felsenstein 1978). From this trimmed alignment, all of the *H. poeyi* haplotypes we found with our new primers remained, with only one additional singleton haplotype collected from Anne Arundel County, Maryland (Appendix S6). Notably, the sequences for *H. poeyi* available from both Cuba and the Dominican Republic fall within Hp01 (Figure 4), indicating that *H. poeyi* is indeed the correct name for this lineage rather than *H. capitosus* , as was discussed in Carman and Packer (1996). There were 19 additional *H. ligatus* haplotypes represented in the public data that were not present in our sample set from NC. Haplotype Hl06 was the most represented haplotype in this dataset with 276 individuals, the bulk of which were collected in Ontario, Canada, in 2009 and Montana, USA, in 2019, possibly skewing the commonality of this haplotype due to sampling effort and highlighting issues of uneven sampling in general among such studies. These findings from larger comparisons confirm what we found in NC—that *H. ligatus* is more genetically diverse and likely has a much larger effective population size compared to *H. poeyi* . The collecting efforts dedicated to *H. ligatus* in Canada resulted in 13 haplotypes that are unique to that region; similarly, our collection efforts uncovered two *H. poeyi* haplotypes unique to NC. In both cases, these may be centers of haplotype diversity, or they may simply be artifacts of sampling bias. The sequences we generated for *H. poeyi* in this study, for example, will more than quadruple the publicly available sequence data for this species, just as 77% of all publicly available *H. ligatus* sequences are from Ontario, Canada (Ratnasingham 2024).

Resolving high *H. poeyi* haplotype diversity in NC highlights the need for greater sampling and sequencing from the Caribbean basin as well as the Gulf Coast states of Alabama, Louisiana, Mississippi, and Texas, USA, further inland from those made by Packer (1999), to ensure that sampling bias did not skew the present results. Higher sampling densities in these Gulf Coast states might reveal previously undetected haplotypes, panmixis, or new centers of diversity. Follow‐up sampling in undersampled areas was also proposed by Packer (1999) such that areas of sympatry and the northern extent of *H. poeyi* could be better resolved. Properly mapping haplotype diversity is necessary to test the hypotheses for the origin of *H. poeyi* presented by Carman and Packer (1996). The presence of high haplotype diversity (Figure 4) and early diverging haplotypes (Appendix S5) in NC is consistent with this location being a center of origin (e.g., Zietkiewicz et al. 2003; O'Loughlin et al. 2008; Valade et al. 2009). If the center of origin pattern in NC is confirmed after additional sampling has been made, this will not support any of the Carman and Packer (1996) hypotheses in which *H. poeyi* was thought to have initially diverged in either Florida, USA (hypothesis i), or Central America (hypotheses ii and iii).

In addition to *H. poeyi* , there have been two other morphologically indistinguishable cryptic species proposed to exist within *H. ligatus* . One of these, *H. townsendi* , has been proposed to be a distinct species present in Mexico (Packer et al. 2016; based on the authors' personal observations). At the time of its description, Cockerell stated that *H. townsendi* was “[a] very distinct species, allied to *H. ligatus* ” (Cockerell 1896). In our analyses, we found that *H. townsendi* sequences from BOLD resolved sister to the clade containing *H. ligatus* and *H. poeyi* (Appendix S5). The other morphologically cryptic *Halictus* species of concern is an unnamed species that has only been collected in the Bentsen‐Rio Grande Valley State Park in Texas by students participating in the BioBus project (Elert 2011). These *Halictus* specimens were sequenced by the Centre for Biodiversity Genomics in Guelph, Ontario, Canada, and uploaded directly to BOLD. When these *Halictus* sp. sequences were analyzed, they resolved sister to a clade containing *H. poeyi* , *H. ligatus* , and *H. townsendi* (Appendix S5A) or sister to *H. townsendi* (Appendix S5B), depending on methodology, and were well supported as a distinct species. Follow‐up work will be needed to properly describe this previously uncharacterized species and better understand its ecology and evolution as it relates to *Halictus* phylogeography.

The *H. ligatus/poeyi* species complex is but one example of an insect lineage that has an important ecological impact yet has not been accurately described due to incomplete resolution of cryptic species. These *Halictus* species therefore afford an important study system for examining the historical biogeography of North America as well as a foundation for future studies, including how changes in land use, pollen resources, and climate drive persistence of these bees in a given region. The description of the new HAL primers makes research questions surrounding these *Halictus* species possible to address. One possible area of research that would be valuable to test with improved molecular identification is the re‐examination of morphological data that aligns with molecular genealogy to find characters that resolve these two *Halictus* species (i.e., reciprocal illumination, Hennig 1966). With proper identification, reciprocal transplantation between *H. ligatus* and *H. poeyi* (as has been done with other halictid species (Boulton and Field 2022)), as well as intraspecific haplotype transplantation, could be conducted to address questions related to species distribution (Via and Lande 1985). While these species have been considered common (Michener and Bennett 1977), the true conservation status of each can now be assessed with detailed molecular identification. Further, geographic areas for priority preservation can be identified based on habitat requirements and genetic diversity (Bickford et al. 2006); from the results of our study, it appears that NC may be such a location. As efforts for improving wild bee monitoring and conservation are increasing (Woodard et al. 2020; Levenson et al. 2024), accurate information about species delimitation and distributions, such as what the HAL primers have provided here, will be critical. Similar techniques should also be applied to other wild bee species.

## Author Contributions

**Hannah K. Levenson:** conceptualization (equal), data curation (equal), investigation (equal), methodology (equal), project administration (lead), resources (equal), visualization (equal), writing – original draft (lead), writing – review and editing (lead). **Luke R. Tembrock:** conceptualization (equal), data curation (supporting), formal analysis (equal), funding acquisition (equal), investigation (equal), methodology (equal), project administration (equal), resources (equal), supervision (equal), visualization (equal), writing – original draft (supporting), writing – review and editing (supporting). **Frida A. Zink:** data curation (equal), formal analysis (equal), investigation (equal), methodology (equal), visualization (equal), writing – original draft (supporting), writing – review and editing (equal). **Kayla A. Mollet:** data curation (equal), formal analysis (equal), investigation (equal), methodology (supporting), visualization (supporting), writing – original draft (supporting), writing – review and editing (supporting). **David R. Tarpy:** conceptualization (equal), funding acquisition (equal), methodology (equal), project administration (equal), resources (equal), supervision (equal), writing – original draft (supporting), writing – review and editing (supporting).

## Conflicts of Interest

The authors declare no conflicts of interest.

## Supporting information


Appendix S1.

## Data Availability

For more detailed data on the specimens and sample collection methods, see Levenson and Tarpy (2023). All sequences generated for this study are available in GenBank under accession numbers PQ576549–PQ576636.
