# Supplementary material for: Redefining the Geographic Distribution of Two Cryptic Halictus (Hymenoptera: Halictidae) Species in the Eastern United States
Source: Ecol Evol. 2025 Jun 20;15(6):e71570. doi: 10.1002/ece3.71570 (PMC12180375; doi:10.1002/ece3.71570)
Supplement: Supplementary file 1 — Appendix S1. [file ECE3-15-e71570-s001.docx]

**Appendices**

Appendix 1. Sanger sequence quality metrics resulting from the use of universal primer set LCO1490 and HCO2198 compared to the new *Halictus* primer set HAL_134_F and HAL_958_R.

| **Primer set** | **Number of *Halictus* sampled** | **Number of samples detected on gel** | **Sequence length (mean ± SD)** | **% of nucleotides per sequence with a Phred quality score of ≥ 40** | | **Non-target sequences** | **Number of samples with useable data** |
| --- | --- | --- | --- | --- | --- | --- | --- |
|  |  |  |  | **Forward sequence (mean ± SD)** | **Reverse sequence (mean ± SD)** |  |  |
| LCO1490 and HCO2198 | 136 | 111 | 643 ± 173 | 49.89% ± 41% | 46.82% ± 41% | 19* | 59 |
| HAL_134_F and HAL_958_R | 99 | 97 | 797 ± 9 | 82.2% ± 11% | 82.17% ± 15% | 0 | 93 |

*These 19 sequences generated using the LCO1490 and HCO2198 primer set produced sequences not matching to *Halictus* when searched against GenBank using BLASTn. Other sequences generated from these primers were probably also the result of nontarget amplification but were of such low quality that they could not be interpreted.

Appendix 2. A Bayesian analysis of CO1 sequence data for all NC, USA sequenced specimens using HAL primers. Outgroups are *Halictus rubicundus* (AF102842.1, JQ266430.1, JQ266430.1, KT1646681.1; accessions from Genbank) as well as *Lasioglossum lustrans* collected at Border Belt (this study). For the sequences generated in this study, the first two letters of each terminal name correspond to a collection location and the last two letters indicate species. Branch labels indicate posterior probability clade support.

**
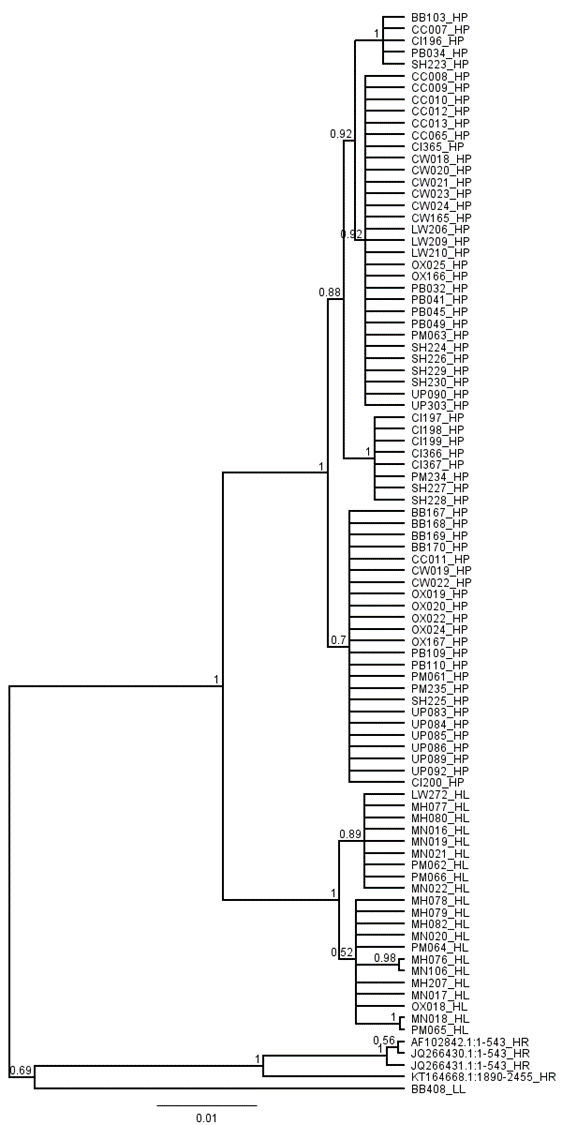
**

Appendix 3. Soil properties at each collection location regressed on longitude. The soil properties are the same as those employed in the Geneland analyses (Figure 3) and include A) average soil moisture, B) average soil temperature, and C) estimated geologic age of soil parent material.

**
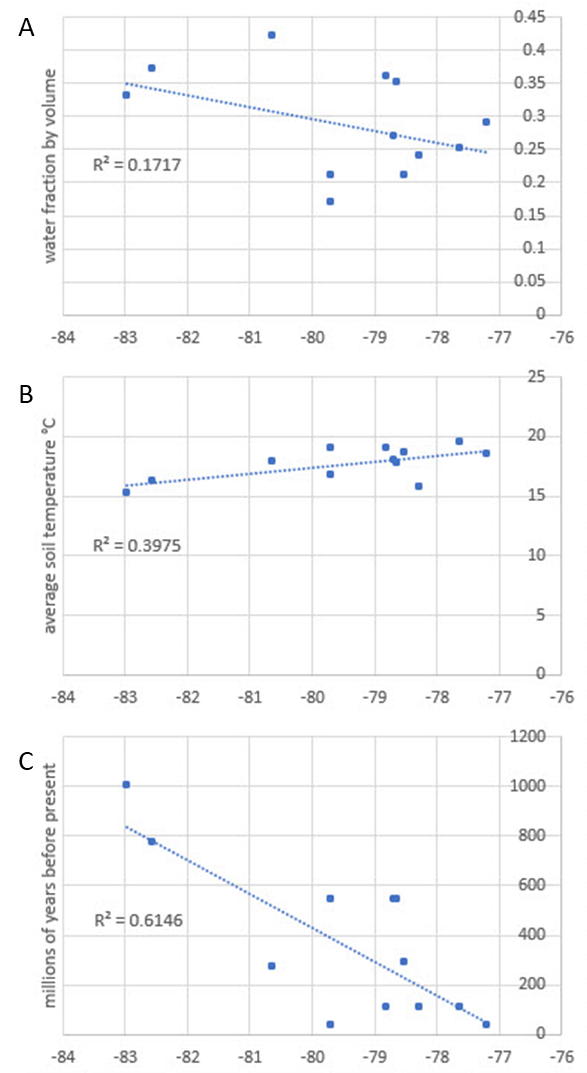
**

Appendix 4. Species delimitation analysis was carried out to determine whether there is any cryptic speciation within NC, USA populations of either *H. ligatus* or *H. poeyi* using CO1 sequence data. A Bayesian tree was used to determine whether any of the monophyletic clades within each species or the outgroup (*H. rubicundus*) was supported as a cryptic species. Each clade tested in each scenario is shown collapsed to the most recent common ancestor and numbered.

**
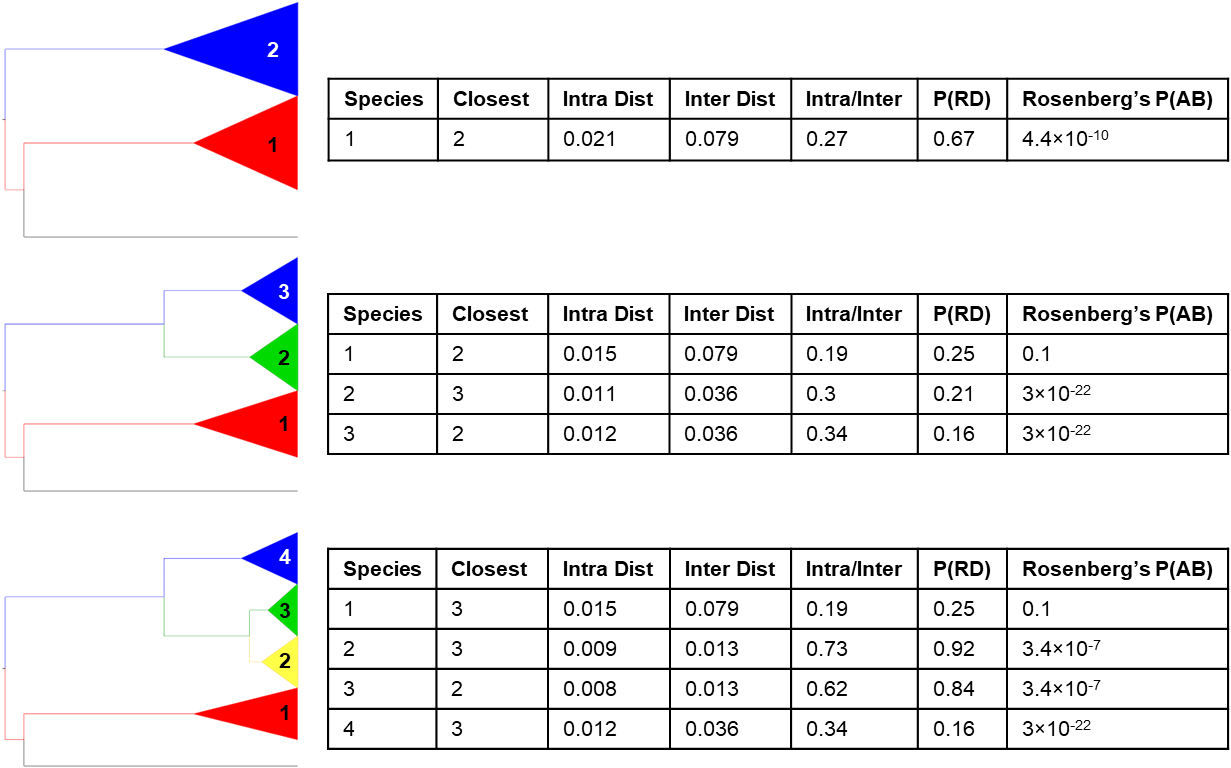
**

**
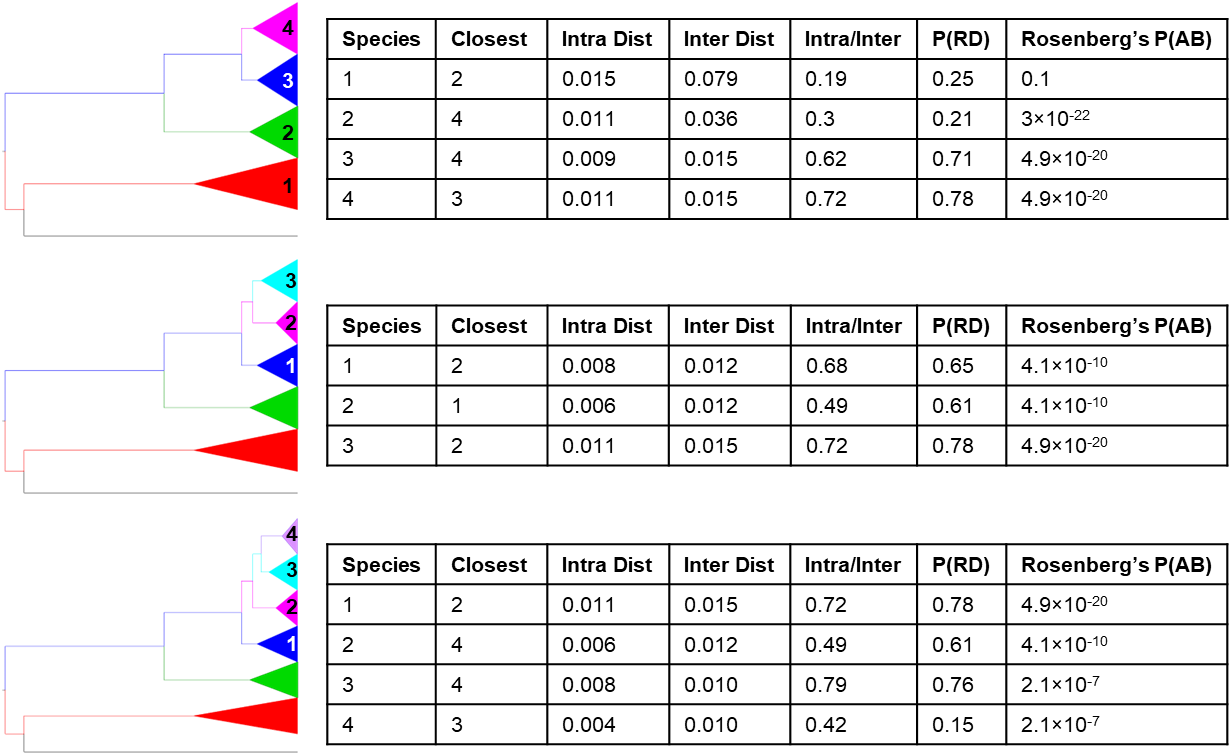
**

Appendix 5. Phylogenetic trees of CO1 sequence data in A) a neighbor-joining tree and B) a Bayesian analysis both generated from combined data from this study and public data mined from BOLD for *H. ligatus*, *H. poeyi*, *H. townsendi*, and *Halictus* sp. from Texas. The total number of nodes was reduced to include single representatives of each unique sequence of *H. ligatus* and *H. poeyi*. Each terminal label corresponds to halplotype designations as presented in Appendix 6. *Lasioglossum lustrans* was used as an outgroup. Branch labels in A represent jackknife node support. Branch labels in B represent posterior probability.

**
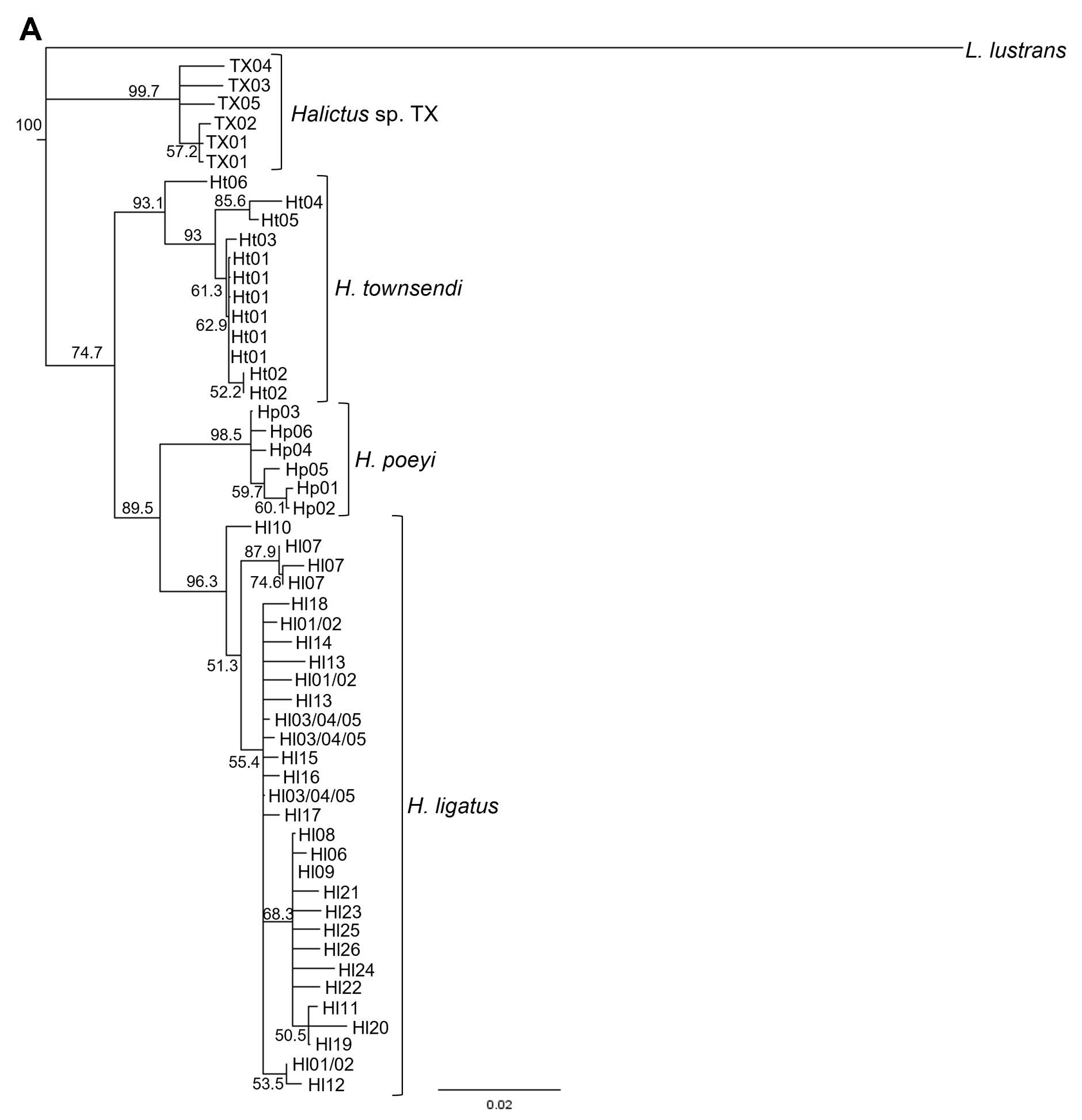
**

**
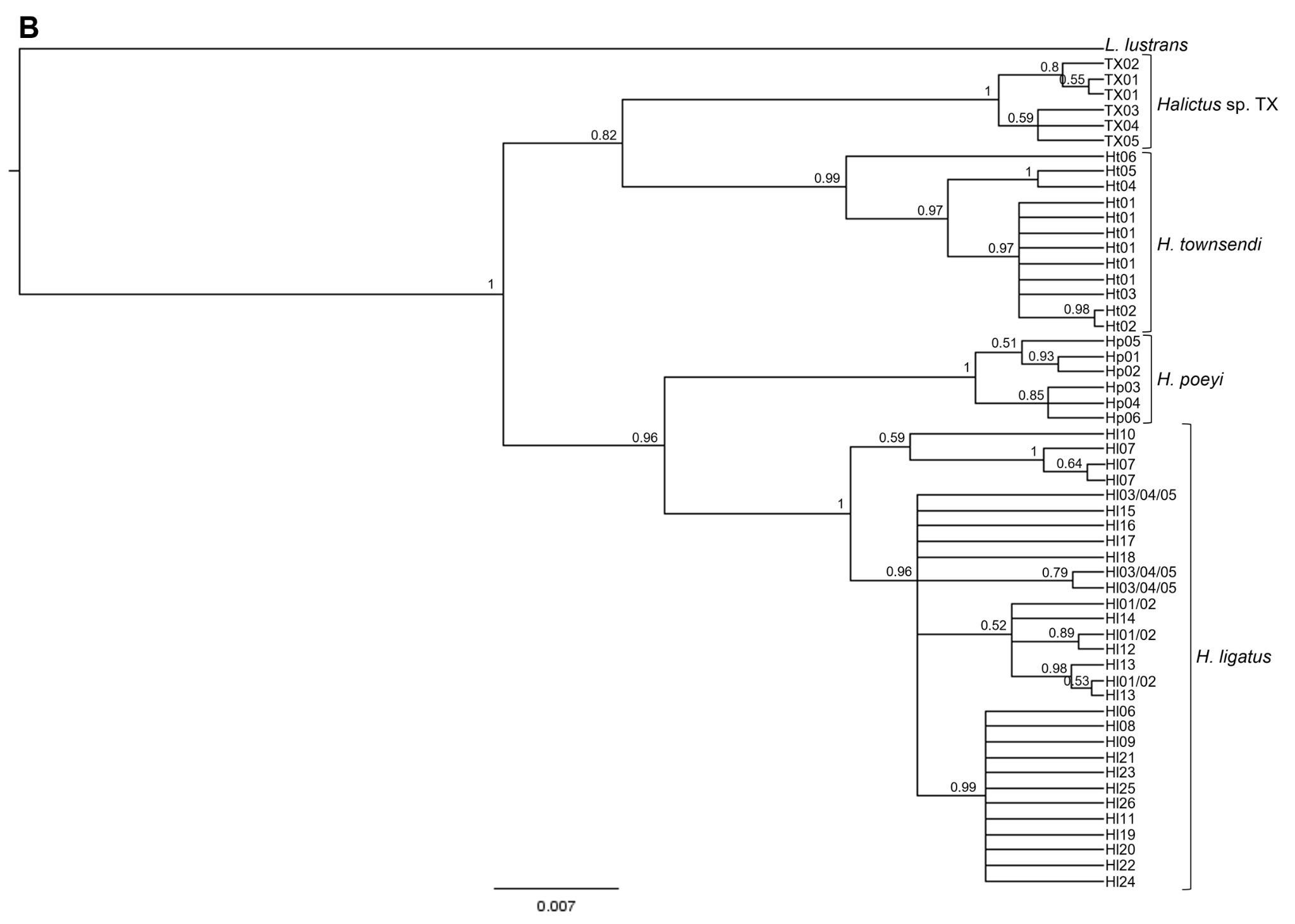
**

Appendix 6. All samples included in analyses in this study.

*Generated for this study

†Mined from BOLD systems

‡Mined from Genbank

| Accession | Species | Location | Haplotype |
| --- | --- | --- | --- |
| BB103* | *Halictus poeyi* | Border Belt Research Station, NC, USA | Hp01 |
| BEECB085-07† | *Halictus poeyi* | Cuba | Hp01 |
| BEECB190-07† | *Halictus poeyi* | Cuba | Hp01 |
| BEECB191-07† | *Halictus poeyi* | Dominican Republic | Hp01 |
| BEECB192-07† | *Halictus poeyi* | Dominican Republic | Hp01 |
| BUSA276-05† | *Halictus poeyi* |  | Hp01 |
| CC007* | *Halictus poeyi* | Central Crops Research Station, NC, USA | Hp01 |
| CI196* | *Halictus poeyi* | Horticultural Crops Research Station, Clinton, NC, USA | Hp01 |
| PB034* | *Halictus poeyi* | Peanut Belt Research Station, NC, USA | Hp01 |
| SH223* | *Halictus poeyi* | Sandhills Research Station, NC, USA | Hp01 |
| BBHYA1329-12† | *Halictus poeyi* | Kissimmee, FL, USA | Hp02 |
| BUSA274-05† | *Halictus poeyi* |  | Hp02 |
| CC008* | *Halictus poeyi* | Central Crops Research Station, NC, USA | Hp02 |
| CC009* | *Halictus poeyi* | Central Crops Research Station, NC, USA | Hp02 |
| CC010* | *Halictus poeyi* | Central Crops Research Station, NC, USA | Hp02 |
| CC012* | *Halictus poeyi* | Central Crops Research Station, NC, USA | Hp02 |
| CC013* | *Halictus poeyi* | Central Crops Research Station, NC, USA | Hp02 |
| CC065* | *Halictus poeyi* | Central Crops Research Station, NC, USA | Hp02 |
| CI365* | *Halictus poeyi* | Horticultural Crops Research Station, Clinton, NC, USA | Hp02 |
| CW018* | *Halictus poeyi* | Caswell Research Station, NC, USA | Hp02 |
| CW020* | *Halictus poeyi* | Caswell Research Station, NC, USA | Hp02 |
| CW021* | *Halictus poeyi* | Caswell Research Station, NC, USA | Hp02 |
| CW023* | *Halictus poeyi* | Caswell Research Station, NC, USA | Hp02 |
| CW024* | *Halictus poeyi* | Caswell Research Station, NC, USA | Hp02 |
| CW165* | *Halictus poeyi* | Caswell Research Station, NC, USA | Hp02 |
| DIAL046-06† | *Halictus poeyi* | PAVO, GA, USA | Hp02 |
| DIAL047-06† | *Halictus poeyi* | PAVO, GA, USA | Hp02 |
| LW206* | *Halictus poeyi* | Lake Wheeler Research Station, NC, USA | Hp02 |
| LW209* | *Halictus poeyi* | Lake Wheeler Research Station, NC, USA | Hp02 |
| LW210* | *Halictus poeyi* | Lake Wheeler Research Station, NC, USA | Hp02 |
| OX025* | *Halictus poeyi* | Oxford Research Station, NC, USA | Hp02 |
| OX166* | *Halictus poeyi* | Oxford Research Station, NC, USA | Hp02 |
| PB032* | *Halictus poeyi* | Peanut Belt Research Station, NC, USA | Hp02 |
| PB041* | *Halictus poeyi* | Peanut Belt Research Station, NC, USA | Hp02 |
| PB045* | *Halictus poeyi* | Peanut Belt Research Station, NC, USA | Hp02 |
| PB049* | *Halictus poeyi* | Peanut Belt Research Station, NC, USA | Hp02 |
| PM063* | *Halictus poeyi* | Piedmont Research Station, NC, USA | Hp02 |
| SH224* | *Halictus poeyi* | Sandhills Research Station, NC, USA | Hp02 |
| SH226* | *Halictus poeyi* | Sandhills Research Station, NC, USA | Hp02 |
| SH229* | *Halictus poeyi* | Sandhills Research Station, NC, USA | Hp02 |
| SH230* | *Halictus poeyi* | Sandhills Research Station, NC, USA | Hp02 |
| UP090* | *Halictus poeyi* | Upper Piedmont Research Station, NC, USA | Hp02 |
| UP303* | *Halictus poeyi* | Upper Piedmont Research Station, NC, USA | Hp02 |
| BB167* | *Halictus poeyi* | Border Belt Research Station, NC, USA | Hp03 |
| BB168* | *Halictus poeyi* | Border Belt Research Station, NC, USA | Hp03 |
| BB169* | *Halictus poeyi* | Border Belt Research Station, NC, USA | Hp03 |
| BB170* | *Halictus poeyi* | Border Belt Research Station, NC, USA | Hp03 |
| BBBEE889-11† | *Halictus poeyi* | Fulton County, GA, USA | Hp03 |
| BBHYA2960-12† | *Halictus poeyi* | Kissimmee, FL, USA | Hp03 |
| BEECB088-07† | *Halictus poeyi* | USA | Hp03 |
| BUSA275-05† | *Halictus poeyi* |  | Hp03 |
| CC011* | *Halictus poeyi* | Central Crops Research Station, NC, USA | Hp03 |
| CW019* | *Halictus poeyi* | Caswell Research Station, NC, USA | Hp03 |
| CW022* | *Halictus poeyi* | Caswell Research Station, NC, USA | Hp03 |
| GBMNB64132-20† | *Halictus poeyi* | University of Alabama Arboretum, Tuscaloosa, AL, USA | Hp03 |
| OX019* | *Halictus poeyi* | Oxford Research Station, NC, USA | Hp03 |
| OX020* | *Halictus poeyi* | Oxford Research Station, NC, USA | Hp03 |
| OX022* | *Halictus poeyi* | Oxford Research Station, NC, USA | Hp03 |
| OX024* | *Halictus poeyi* | Oxford Research Station, NC, USA | Hp03 |
| OX025* | *Halictus poeyi* | Oxford Research Station, NC, USA | Hp03 |
| OX167* | *Halictus poeyi* | Oxford Research Station, NC, USA | Hp03 |
| PB109* | *Halictus poeyi* | Peanut Belt Research Station, NC, USA | Hp03 |
| PB110* | *Halictus poeyi* | Peanut Belt Research Station, NC, USA | Hp03 |
| PM061* | *Halictus poeyi* | Piedmont Research Station, NC, USA | Hp03 |
| PM235* | *Halictus poeyi* | Piedmont Research Station, NC, USA | Hp03 |
| SH225* | *Halictus poeyi* | Sandhills Research Station, NC, USA | Hp03 |
| UP083* | *Halictus poeyi* | Upper Piedmont Research Station, NC, USA | Hp03 |
| UP084* | *Halictus poeyi* | Upper Piedmont Research Station, NC, USA | Hp03 |
| UP085* | *Halictus poeyi* | Upper Piedmont Research Station, NC, USA | Hp03 |
| UP086* | *Halictus poeyi* | Upper Piedmont Research Station, NC, USA | Hp03 |
| UP089* | *Halictus poeyi* | Upper Piedmont Research Station, NC, USA | Hp03 |
| UP092* | *Halictus poeyi* | Upper Piedmont Research Station, NC, USA | Hp03 |
| CI200* | *Halictus poeyi* | Horticultural Crops Research Station, Clinton, NC, USA | Hp04 |
| CI197* | *Halictus poeyi* | Horticultural Crops Research Station, Clinton, NC, USA | Hp05 |
| CI198* | *Halictus poeyi* | Horticultural Crops Research Station, Clinton, NC, USA | Hp05 |
| CI199* | *Halictus poeyi* | Horticultural Crops Research Station, Clinton, NC, USA | Hp05 |
| CI366* | *Halictus poeyi* | Horticultural Crops Research Station, Clinton, NC, USA | Hp05 |
| CI367* | *Halictus poeyi* | Horticultural Crops Research Station, Clinton, NC, USA | Hp05 |
| PM234* | *Halictus poeyi* | Piedmont Research Station, NC, USA | Hp05 |
| SH227* | *Halictus poeyi* | Sandhills Research Station, NC, USA | Hp05 |
| SH228* | *Halictus poeyi* | Sandhills Research Station, NC, USA | Hp05 |
| BUSA178-05† | *Halictus poeyi* | Anne Arundel, MD, USA | Hp06 |
| BEO058-04† | *Halictus ligatus* |  | Hl01/02 |
| GBMIN77148-17† | *Halictus ligatus* | Lexington, KY, USA | Hl01/02 |
| HYMBB050-09† | *Halictus ligatus* | El Paso County, CO, USA | Hl01/02 |
| JSHYN425-11† | *Halictus ligatus* | Leeds and Grenville, ON, CA | Hl01/02 |
| LW272* | *Halictus ligatus* | Lake Wheeler Research Station, NC, USA | Hl01 |
| MH078* | *Halictus ligatus* | Mountain Horticulture Research Farm & Extension, NC, USA | Hl02 |
| MH079* | *Halictus ligatus* | Mountain Horticulture Research Farm & Extension, NC, USA | Hl02 |
| MH082* | *Halictus ligatus* | Mountain Horticulture Research Farm & Extension, NC, USA | Hl02 |
| MN020* | *Halictus ligatus* | Mountain Research Station, NC, USA | Hl02 |
| MPGA3037-19† | *Halictus ligatus* | Florence, MT, USA | Hl01/02 |
| MPGE191-19† | *Halictus ligatus* | Florence, MT, USA | Hl01/02 |
| MPGF145-19† | *Halictus ligatus* | Florence, MT, USA | Hl01/02 |
| MPGF1163-19† | *Halictus ligatus* | Florence, MT, USA | Hl01/02 |
| MPGO666-19† | *Halictus ligatus* | Florence, MT, USA | Hl01/02 |
| MPGO684-19† | *Halictus ligatus* | Florence, MT, USA | Hl01/02 |
| MPGO4002-19† | *Halictus ligatus* | Florence, MT, USA | Hl01/02 |
| MPGQ807-19† | *Halictus ligatus* | Florence, MT, USA | Hl01/02 |
| MPGT1589-19† | *Halictus ligatus* | Florence, MT, USA | Hl01/02 |
| PM064* | *Halictus ligatus* | Piedmont Research Station, NC, USA | Hl02 |
| TTHYW049-08† | *Halictus ligatus* | Grasslands National Park, SK, CA | Hl01/02 |
| UAIC533-21† | *Halictus ligatus* | Tucson, AZ, USA | Hl01/02 |
| UAIC1847-22† | *Halictus ligatus* | Tucson, AZ, USA | Hl01/02 |
| UAIC1856-22† | *Halictus ligatus* | Tucson, AZ, USA | Hl01/02 |
| UAIC1858-22† | *Halictus ligatus* | Tucson, AZ, USA | Hl01/02 |
| UAIC1875-22† | *Halictus ligatus* | Tucson, AZ, USA | Hl01/02 |
| UPOLB264-09† | *Halictus ligatus* | Cambridge, ON, CA | Hl01/02 |
| UPOLB658-09† | *Halictus ligatus* | Cambridge, ON, CA | Hl01/02 |
| UPOLB756-09† | *Halictus ligatus* | Cambridge, ON, CA | Hl01/02 |
| UPOLC008-09† | *Halictus ligatus* | Cambridge, ON, CA | Hl01/02 |
| UPOLL038-09† | *Halictus ligatus* | Cambridge, ON, CA | Hl01/02 |
| UPOLL040-09† | *Halictus ligatus* | Cambridge, ON, CA | Hl01/02 |
| UPOLL044-09† | *Halictus ligatus* | Cambridge, ON, CA | Hl01/02 |
| UPOLL049-09† | *Halictus ligatus* | Cambridge, ON, CA | Hl01/02 |
| UPOLL061-09† | *Halictus ligatus* | Cambridge, ON, CA | Hl01/02 |
| UPOLL063-09† | *Halictus ligatus* | Cambridge, ON, CA | Hl01/02 |
| UPOLL076-09† | *Halictus ligatus* | Cambridge, ON, CA | Hl01/02 |
| UPOLL089-09† | *Halictus ligatus* | Cambridge, ON, CA | Hl01/02 |
| UPOLL111-09† | *Halictus ligatus* | Cambridge, ON, CA | Hl01/02 |
| UPOLL161-09† | *Halictus ligatus* | Cambridge, ON, CA | Hl01/02 |
| UPOLL171-09† | *Halictus ligatus* | Cambridge, ON, CA | Hl01/02 |
| UPOLL173-09† | *Halictus ligatus* | Cambridge, ON, CA | Hl01/02 |
| UPOLL409-09† | *Halictus ligatus* | Guelph, ON, CA | Hl01/02 |
| UPOLL467-09† | *Halictus ligatus* | Cambridge, ON, CA | Hl01/02 |
| UPOLL472-09† | *Halictus ligatus* | Cambridge, ON, CA | Hl01/02 |
| UPOLL479-09† | *Halictus ligatus* | Cambridge, ON, CA | Hl01/02 |
| UPOLL483-09† | *Halictus ligatus* | Cambridge, ON, CA | Hl01/02 |
| UPOLL490-09† | *Halictus ligatus* | Cambridge, ON, CA | Hl01/02 |
| UPOLL531-09† | *Halictus ligatus* | Guelph, ON, CA | Hl01/02 |
| UPOLL533-09† | *Halictus ligatus* | Guelph, ON, CA | Hl01/02 |
| UPOLL660-09† | *Halictus ligatus* | Cambridge, ON, CA | Hl01/02 |
| UPOLL675-09† | *Halictus ligatus* | Cambridge, ON, CA | Hl01/02 |
| UPOLL718-09† | *Halictus ligatus* | Guelph, ON, CA | Hl01/02 |
| UPOLL739-09† | *Halictus ligatus* | Guelph, ON, CA | Hl01/02 |
| UPOLL803-09† | *Halictus ligatus* | Cambridge, ON, CA | Hl01/02 |
| UPOLL804-09† | *Halictus ligatus* | Cambridge, ON, CA | Hl01/02 |
| UPOLL930-09† | *Halictus ligatus* | Guelph, ON, CA | Hl01/02 |
| BANT102-10† | *Halictus ligatus* | Toronto, ON, CA | Hl03/04/05 |
| BBBEE882-11† | *Halictus ligatus* | Atlanta, GA, USA | Hl03/04/05 |
| BBBEE890-11† | *Halictus ligatus* | Atlanta, GA, USA | Hl03/04/05 |
| BEECB075-07† | *Halictus ligatus* | Ontario, CA | Hl03/04/05 |
| BEECB239-07† | *Halictus ligatus* |  | Hl03/04/05 |
| BOFTW1320-15† | *Halictus ligatus* | Greenlee County, AZ, USA | Hl03/04/05 |
| BOWAU146-11† | *Halictus ligatus* | Spring Valley, CA, USA | Hl03/04/05 |
| GBMIN77146-11† | *Halictus ligatus* | Lexington, KY, USA | Hl03/04/05 |
| GBMIN77147-17† | *Halictus ligatus* | Lexington, KY, USA | Hl03/04/05 |
| MH076* | *Halictus ligatus* | Mountain Horticulture Research Farm & Extension, NC, USA | Hl03 |
| MN106* | *Halictus ligatus* | Mountain Research Station, NC, USA | Hl03 |
| MH207* | *Halictus ligatus* | Mountain Horticulture Research Farm & Extension, NC, USA | Hl04 |
| MN017* | *Halictus ligatus* | Mountain Research Station, NC, USA | Hl04 |
| MPG2048-21† | *Halictus ligatus* | Florence, MT, USA | Hl03/04/05 |
| MPGO661-19† | *Halictus ligatus* | Florence, MT, USA | Hl03/04/05 |
| MPGO3947-19† | *Halictus ligatus* | Florence, MT, USA | Hl03/04/05 |
| NCCC648-11† | *Halictus ligatus* | Kingston, ON, CA | Hl03/04/05 |
| OPPOE264-17† | *Halictus ligatus* | Warsaw, ON, CA | Hl03/04/05 |
| OPPQM231-17† | *Halictus ligatus* | Thorold, ON, CA | Hl03/04/05 |
| OX018* | *Halictus ligatus* | Oxford Research Station, NC, USA | Hl04 |
| MH077* | *Halictus ligatus* | Mountain Horticulture Research Farm & Extension, NC, USA | Hl05 |
| MH080* | *Halictus ligatus* | Mountain Horticulture Research Farm & Extension, NC, USA | Hl05 |
| MN016* | *Halictus ligatus* | Mountain Research Station, NC, USA | Hl05 |
| MN019* | *Halictus ligatus* | Mountain Research Station, NC, USA | Hl05 |
| MN021* | *Halictus ligatus* | Mountain Research Station, NC, USA | Hl05 |
| PM062* | *Halictus ligatus* | Piedmont Research Station, NC, USA | Hl05 |
| PM066* | *Halictus ligatus* | Piedmont Research Station, NC, USA | Hl05 |
| RRSSC4975-75† | *Halictus ligatus* | Cambridge, ON, CA | Hl03/04/05 |
| UAIC254-21† | *Halictus ligatus* | Tucson, AZ, USA | Hl03/04/05 |
| UAIC1061-21† | *Halictus ligatus* | Pima County, AZ, USA | Hl03/04/05 |
| UAIC1062-21† | *Halictus ligatus* | Pima County, AZ, USA | Hl03/04/05 |
| UAIC1852-22† | *Halictus ligatus* | Tucson, AZ, USA | Hl03/04/05 |
| UAIC1862-22† | *Halictus ligatus* | Tucson, AZ, USA | Hl03/04/05 |
| UPOLB002-09† | *Halictus ligatus* | Cambridge, ON, CA | Hl03/04/05 |
| UPOLB114-09† | *Halictus ligatus* | Cambridge, ON, CA | Hl03/04/05 |
| UPOLB136-09† | *Halictus ligatus* | Cambridge, ON, CA | Hl03/04/05 |
| UPOLB137-09† | *Halictus ligatus* | Cambridge, ON, CA | Hl03/04/05 |
| UPOLB155-09† | *Halictus ligatus* | Cambridge, ON, CA | Hl03/04/05 |
| UPOLB163-09† | *Halictus ligatus* | Cambridge, ON, CA | Hl03/04/05 |
| UPOLB165-09† | *Halictus ligatus* | Cambridge, ON, CA | Hl03/04/05 |
| UPOLB174-09† | *Halictus ligatus* | Guelph, ON, CA | Hl03/04/05 |
| UPOLB198-09† | *Halictus ligatus* | Guelph, ON, CA | Hl03/04/05 |
| UPOLB199-09† | *Halictus ligatus* | Guelph, ON, CA | Hl03/04/05 |
| UPOLB259-09† | *Halictus ligatus* | Cambridge, ON, CA | Hl03/04/05 |
| UPOLB285-09† | *Halictus ligatus* | Guelph, ON, CA | Hl03/04/05 |
| UPOLB315-09† | *Halictus ligatus* | Guelph, ON, CA | Hl03/04/05 |
| UPOLB330-09† | *Halictus ligatus* | Cambridge, ON, CA | Hl03/04/05 |
| UPOLB352-09† | *Halictus ligatus* | Guelph, ON, CA | Hl03/04/05 |
| UPOLB429-09† | *Halictus ligatus* | Guelph, ON, CA | Hl03/04/05 |
| UPOLB451-09† | *Halictus ligatus* | Kitchener, ON, CA | Hl03/04/05 |
| UPOLB460-09† | *Halictus ligatus* | Cambridge, ON, CA | Hl03/04/05 |
| UPOLB466-09† | *Halictus ligatus* | Guelph, ON, CA | Hl03/04/05 |
| UPOLB902-09† | *Halictus ligatus* | Cambridge, ON, CA | Hl03/04/05 |
| UPOLB949-09† | *Halictus ligatus* | Kitchener, ON, CA | Hl03/04/05 |
| UPOLC005-09† | *Halictus ligatus* | Cambridge, ON, CA | Hl03/04/05 |
| UPOLL070-09† | *Halictus ligatus* | Cambridge, ON, CA | Hl03/04/05 |
| UPOLL110-09† | *Halictus ligatus* | Cambridge, ON, CA | Hl03/04/05 |
| UPOLL159-09† | *Halictus ligatus* | Cambridge, ON, CA | Hl03/04/05 |
| UPOLL394-09† | *Halictus ligatus* | Guelph, ON, CA | Hl03/04/05 |
| UPOLL435-09† | *Halictus ligatus* | Cambridge, ON, CA | Hl03/04/05 |
| UPOLL444-09† | *Halictus ligatus* | Cambridge, ON, CA | Hl03/04/05 |
| UPOLL448-09† | *Halictus ligatus* | Cambridge, ON, CA | Hl03/04/05 |
| UPOLL466-09† | *Halictus ligatus* | Cambridge, ON, CA | Hl03/04/05 |
| UPOLL471-09† | *Halictus ligatus* | Cambridge, ON, CA | Hl03/04/05 |
| UPOLL488-09† | *Halictus ligatus* | Cambridge, ON, CA | Hl03/04/05 |
| UPOLL501-09† | *Halictus ligatus* | Cambridge, ON, CA | Hl03/04/05 |
| UPOLL558-09† | *Halictus ligatus* | Guelph, ON, CA | Hl03/04/05 |
| UPOLL594-09† | *Halictus ligatus* | Cambridge, ON, CA | Hl03/04/05 |
| UPOLL605-09† | *Halictus ligatus* | Cambridge, ON, CA | Hl03/04/05 |
| UPOLL630-09† | *Halictus ligatus* | Guelph, ON, CA | Hl03/04/05 |
| UPOLL639-09† | *Halictus ligatus* | Guelph, ON, CA | Hl03/04/05 |
| UPOLL669-09† | *Halictus ligatus* | Cambridge, ON, CA | Hl03/04/05 |
| UPOLL672-09† | *Halictus ligatus* | Cambridge, ON, CA | Hl03/04/05 |
| UPOLL721-09† | *Halictus ligatus* | Guelph, ON, CA | Hl03/04/05 |
| UPOLL766-09† | *Halictus ligatus* | Cambridge, ON, CA | Hl03/04/05 |
| UPOLL773-09† | *Halictus ligatus* | Cambridge, ON, CA | Hl03/04/05 |
| UPOLL776-09† | *Halictus ligatus* | Cambridge, ON, CA | Hl03/04/05 |
| UPOLL789-09† | *Halictus ligatus* | Guelph, ON, CA | Hl03/04/05 |
| UPOLL816-09† | *Halictus ligatus* | Cambridge, ON, CA | Hl03/04/05 |
| UPOLL913-09† | *Halictus ligatus* | Cambridge, ON, CA | Hl03/04/05 |
| UPOLL941-09† | *Halictus ligatus* | Waterloo, ON, CA | Hl03/04/05 |
| BANT159-10† | *Halictus ligatus* | Toronto, ON, CA | Hl06 |
| BANT160-10† | *Halictus ligatus* | Toronto, ON, CA | Hl06 |
| BANT166-10† | *Halictus ligatus* | Toronto, ON, CA | Hl06 |
| BCII294-11† | *Halictus ligatus* | Okanagan-Similkameen Reg. Dist., BC, CA | Hl06 |
| BCII392-11† | *Halictus ligatus* | Toronto, ON, CA | Hl06 |
| BCLRB020-08† | *Halictus ligatus* | Okanagan-Similkameen Reg. Dist., BC, CA | Hl06 |
| BCLRB061-08† | *Halictus ligatus* | Okanagan-Similkameen Reg. Dist., BC, CA | Hl06 |
| BCLRB065-08† | *Halictus ligatus* | Okanagan-Similkameen Reg. Dist., BC, CA | Hl06 |
| BCLRB658-10† | *Halictus ligatus* | Okanagan-Similkameen Reg. Dist., BC, CA | Hl06 |
| BCLRB676-10† | *Halictus ligatus* | Okanagan-Similkameen Reg. Dist., BC, CA | Hl06 |
| BCLRB679-10† | *Halictus ligatus* | Okanagan-Similkameen Reg. Dist., BC, CA | Hl06 |
| BCLRB680-10† | *Halictus ligatus* | Okanagan-Similkameen Reg. Dist., BC, CA | Hl06 |
| BCLRB681-10† | *Halictus ligatus* | Okanagan-Similkameen Reg. Dist., BC, CA | Hl06 |
| BEECB076-07† | *Halictus ligatus* | Ontario, CA | Hl06 |
| BEEMH035-06† | *Halictus ligatus* | Guelph, ON, CA | Hl06 |
| BEEMH036-06† | *Halictus ligatus* | Guelph, ON, CA | Hl06 |
| BEEMH292-06† | *Halictus ligatus* | Guelph, ON, CA | Hl06 |
| BEO059-04† | *Halictus ligatus* |  | Hl06 |
| BEO060-04† | *Halictus ligatus* |  | Hl06 |
| BUSA101-05† | *Halictus ligatus* | East Hartford, CT, USA | Hl06 |
| BUSA102-05† | *Halictus ligatus* | East Hartford, CT, USA | Hl06 |
| BUSA172-05† | *Halictus ligatus* | Capon Bridge, WV, USA | Hl06 |
| BUSA175-05† | *Halictus ligatus* | Hampshire, WV, USA | Hl06 |
| BUSA442-12† | *Halictus ligatus* | Wallowa, OR, USA | Hl06 |
| BWTWO1265-10† | *Halictus ligatus* | Toronto, ON, CA | Hl06 |
| DIAL174-06† | *Halictus ligatus* | Minnetonka, MN, USA | Hl06 |
| HCBNS211-03† | *Halictus ligatus* | Avonport, NS, CA | Hl06 |
| HCBNS213-03† | *Halictus ligatus* | Upper Canard, NS, CA | Hl06 |
| HYAZ086-09† | *Halictus ligatus* | Yuma County, AZ, USA | Hl06 |
| JSHYO902-11† | *Halictus ligatus* | Leeds and Grenville, ON, CA | Hl06 |
| LFBC1699-18† | *Halictus ligatus* | Kamloops, BC, CA | Hl06 |
| MN018* | *Halictus ligatus* | Mountain Research Station, NC, USA | Hl06 |
| MPG1973-21† | *Halictus ligatus* | Florence, MT, USA | Hl06 |
| MPG2067-21† | *Halictus ligatus* | Florence, MT, USA | Hl06 |
| MPGA2369-19† | *Halictus ligatus* | Florence, MT, USA | Hl06 |
| MPGA2370-19† | *Halictus ligatus* | Florence, MT, USA | Hl06 |
| MPGB462-19† | *Halictus ligatus* | Florence, MT, USA | Hl06 |
| MPGB463-19† | *Halictus ligatus* | Florence, MT, USA | Hl06 |
| MPGC1177-19† | *Halictus ligatus* | Florence, MT, USA | Hl06 |
| MPGD628-19† | *Halictus ligatus* | Florence, MT, USA | Hl06 |
| MPGD5231-19† | *Halictus ligatus* | Florence, MT, USA | Hl06 |
| MPGE729-19† | *Halictus ligatus* | Florence, MT, USA | Hl06 |
| MPGE735-19† | *Halictus ligatus* | Florence, MT, USA | Hl06 |
| MPGE1951-19† | *Halictus ligatus* | Florence, MT, USA | Hl06 |
| MPGE1954-19† | *Halictus ligatus* | Florence, MT, USA | Hl06 |
| MPGF144-19† | *Halictus ligatus* | Florence, MT, USA | Hl06 |
| MPGF148-19† | *Halictus ligatus* | Florence, MT, USA | Hl06 |
| MPGF196-19† | *Halictus ligatus* | Florence, MT, USA | Hl06 |
| MPGF1108-19† | *Halictus ligatus* | Florence, MT, USA | Hl06 |
| MPGF1110-19† | *Halictus ligatus* | Florence, MT, USA | Hl06 |
| MPGF1129-19† | *Halictus ligatus* | Florence, MT, USA | Hl06 |
| MPGF1130-19† | *Halictus ligatus* | Florence, MT, USA | Hl06 |
| MPGF1131-19† | *Halictus ligatus* | Florence, MT, USA | Hl06 |
| MPGF1165-19† | *Halictus ligatus* | Florence, MT, USA | Hl06 |
| MPGF1167-19† | *Halictus ligatus* | Florence, MT, USA | Hl06 |
| MPGF1727-19† | *Halictus ligatus* | Florence, MT, USA | Hl06 |
| MPGF1761-19† | *Halictus ligatus* | Florence, MT, USA | Hl06 |
| MPGG180-19† | *Halictus ligatus* | Florence, MT, USA | Hl06 |
| MPGG181-19† | *Halictus ligatus* | Florence, MT, USA | Hl06 |
| MPGG184-19† | *Halictus ligatus* | Florence, MT, USA | Hl06 |
| MPGG1845-19† | *Halictus ligatus* | Florence, MT, USA | Hl06 |
| MPGG1919-19† | *Halictus ligatus* | Florence, MT, USA | Hl06 |
| MPGG1940-19† | *Halictus ligatus* | Florence, MT, USA | Hl06 |
| MPGG1952-19† | *Halictus ligatus* | Florence, MT, USA | Hl06 |
| MPGG1953-19† | *Halictus ligatus* | Florence, MT, USA | Hl06 |
| MPGG1954-19† | *Halictus ligatus* | Florence, MT, USA | Hl06 |
| MPGG1957-19† | *Halictus ligatus* | Florence, MT, USA | Hl06 |
| MPGG2033-19† | *Halictus ligatus* | Florence, MT, USA | Hl06 |
| MPGG2034-19† | *Halictus ligatus* | Florence, MT, USA | Hl06 |
| MPGG2034-19† | *Halictus ligatus* | Florence, MT, USA | Hl06 |
| MPGH191-19† | *Halictus ligatus* | Florence, MT, USA | Hl06 |
| MPGH2473-19† | *Halictus ligatus* | Florence, MT, USA | Hl06 |
| MPGJ1779-19† | *Halictus ligatus* | Florence, MT, USA | Hl06 |
| MPGK1786-19† | *Halictus ligatus* | Florence, MT, USA | Hl06 |
| MPGK1787-19† | *Halictus ligatus* | Florence, MT, USA | Hl06 |
| MPGK1854-19† | *Halictus ligatus* | Florence, MT, USA | Hl06 |
| MPGK1855-19† | *Halictus ligatus* | Florence, MT, USA | Hl06 |
| MPGK1859-19† | *Halictus ligatus* | Florence, MT, USA | Hl06 |
| MPGK2298-19† | *Halictus ligatus* | Florence, MT, USA | Hl06 |
| MPGK2300-19† | *Halictus ligatus* | Florence, MT, USA | Hl06 |
| MPGK2301-19† | *Halictus ligatus* | Florence, MT, USA | Hl06 |
| MPGL206-19† | *Halictus ligatus* | Florence, MT, USA | Hl06 |
| MPGO652-19† | *Halictus ligatus* | Florence, MT, USA | Hl06 |
| MPGO685-19† | *Halictus ligatus* | Florence, MT, USA | Hl06 |
| MPGO3949-19† | *Halictus ligatus* | Florence, MT, USA | Hl06 |
| MPGO3993-19† | *Halictus ligatus* | Florence, MT, USA | Hl06 |
| MPGP1425-19† | *Halictus ligatus* | Florence, MT, USA | Hl06 |
| MPGQ808-19† | *Halictus ligatus* | Florence, MT, USA | Hl06 |
| MPGS903-19† | *Halictus ligatus* | Florence, MT, USA | Hl06 |
| MPGS912-19† | *Halictus ligatus* | Florence, MT, USA | Hl06 |
| MPGS916-19† | *Halictus ligatus* | Florence, MT, USA | Hl06 |
| MPGT1577-19† | *Halictus ligatus* | Florence, MT, USA | Hl06 |
| NCCC651-11† | *Halictus ligatus* | Ontario, CA | Hl06 |
| OPPOI290-17† | *Halictus ligatus* | Warsaw, ON, CA | Hl06 |
| OPPOI292-17† | *Halictus ligatus* | Warsaw, ON, CA | Hl06 |
| PREXP042-14† | *Halictus ligatus* | Guelph, ON, CA | Hl06 |
| PM065* | *Halictus ligatus* | Piedmont Research Station, NC, USA | Hl06 |
| RRSSC4977-15† | *Halictus ligatus* | Cambridge, ON, CA | Hl06 |
| UAIC1948-23† | *Halictus ligatus* | Pima County, AZ, USA | Hl06 |
| UPOLB023-09† | *Halictus ligatus* | Guelph, ON, CA | Hl06 |
| UPOLB037-09† | *Halictus ligatus* | Cambridge, ON, CA | Hl06 |
| UPOLB080-09† | *Halictus ligatus* | Cambridge, ON, CA | Hl06 |
| UPOLB103-09† | *Halictus ligatus* | Waterloo, ON, CA | Hl06 |
| UPOLB107-09† | *Halictus ligatus* | Cambridge, ON, CA | Hl06 |
| UPOLB109-09† | *Halictus ligatus* | Cambridge, ON, CA | Hl06 |
| UPOLB131-09† | *Halictus ligatus* | Cambridge, ON, CA | Hl06 |
| UPOLB132-09† | *Halictus ligatus* | Cambridge, ON, CA | Hl06 |
| UPOLB133-09† | *Halictus ligatus* | Cambridge, ON, CA | Hl06 |
| UPOLB134-09† | *Halictus ligatus* | Cambridge, ON, CA | Hl06 |
| UPOLB135-09† | *Halictus ligatus* | Cambridge, ON, CA | Hl06 |
| UPOLB138-09† | *Halictus ligatus* | Cambridge, ON, CA | Hl06 |
| UPOLB156-09† | *Halictus ligatus* | Cambridge, ON, CA | Hl06 |
| UPOLB157-09† | *Halictus ligatus* | Cambridge, ON, CA | Hl06 |
| UPOLB158-09† | *Halictus ligatus* | Cambridge, ON, CA | Hl06 |
| UPOLB159-09† | *Halictus ligatus* | Cambridge, ON, CA | Hl06 |
| UPOLB172-09† | *Halictus ligatus* | Guelph, ON, CA | Hl06 |
| UPOLB240-09† | *Halictus ligatus* | Guelph, ON, CA | Hl06 |
| UPOLB284-09† | *Halictus ligatus* | Guelph, ON, CA | Hl06 |
| UPOLB291-09† | *Halictus ligatus* | Guelph, ON, CA | Hl06 |
| UPOLB324-09† | *Halictus ligatus* | Cambridge, ON, CA | Hl06 |
| UPOLB325-09† | *Halictus ligatus* | Cambridge, ON, CA | Hl06 |
| UPOLB326-09† | *Halictus ligatus* | Cambridge, ON, CA | Hl06 |
| UPOLB348-09† | *Halictus ligatus* | Guelph, ON, CA | Hl06 |
| UPOLB361-09† | *Halictus ligatus* | Waterloo, ON, CA | Hl06 |
| UPOLB397-09† | *Halictus ligatus* | Cambridge, ON, CA | Hl06 |
| UPOLB430-09† | *Halictus ligatus* | Guelph, ON, CA | Hl06 |
| UPOLB467-09† | *Halictus ligatus* | Guelph, ON, CA | Hl06 |
| UPOLB674-09† | *Halictus ligatus* | Cambridge, ON, CA | Hl06 |
| UPOLB676-09† | *Halictus ligatus* | Cambridge, ON, CA | Hl06 |
| UPOLB677-09† | *Halictus ligatus* | Cambridge, ON, CA | Hl06 |
| UPOLL005-09† | *Halictus ligatus* | Cambridge, ON, CA | Hl06 |
| UPOLL007-09† | *Halictus ligatus* | Cambridge, ON, CA | Hl06 |
| UPOLL010-09† | *Halictus ligatus* | Cambridge, ON, CA | Hl06 |
| UPOLL014-09† | *Halictus ligatus* | Cambridge, ON, CA | Hl06 |
| UPOLL017-09† | *Halictus ligatus* | Guelph, ON, CA | Hl06 |
| UPOLL039-09† | *Halictus ligatus* | Cambridge, ON, CA | Hl06 |
| UPOLL043-09† | *Halictus ligatus* | Cambridge, ON, CA | Hl06 |
| UPOLL046-09† | *Halictus ligatus* | Cambridge, ON, CA | Hl06 |
| UPOLL050-09† | *Halictus ligatus* | Cambridge, ON, CA | Hl06 |
| UPOLL051-09† | *Halictus ligatus* | Cambridge, ON, CA | Hl06 |
| UPOLL052-09† | *Halictus ligatus* | Cambridge, ON, CA | Hl06 |
| UPOLL057-09† | *Halictus ligatus* | Cambridge, ON, CA | Hl06 |
| UPOLL058-09† | *Halictus ligatus* | Cambridge, ON, CA | Hl06 |
| UPOLL059-09† | *Halictus ligatus* | Cambridge, ON, CA | Hl06 |
| UPOLL062-09† | *Halictus ligatus* | Cambridge, ON, CA | Hl06 |
| UPOLL065-09† | *Halictus ligatus* | Cambridge, ON, CA | Hl06 |
| UPOLL068-09† | *Halictus ligatus* | Cambridge, ON, CA | Hl06 |
| UPOLL069-09† | *Halictus ligatus* | Cambridge, ON, CA | Hl06 |
| UPOLL075-09† | *Halictus ligatus* | Cambridge, ON, CA | Hl06 |
| UPOLL079-09† | *Halictus ligatus* | Cambridge, ON, CA | Hl06 |
| UPOLL081-09† | *Halictus ligatus* | Cambridge, ON, CA | Hl06 |
| UPOLL082-09† | *Halictus ligatus* | Cambridge, ON, CA | Hl06 |
| UPOLL083-09† | *Halictus ligatus* | Cambridge, ON, CA | Hl06 |
| UPOLL085-09† | *Halictus ligatus* | Cambridge, ON, CA | Hl06 |
| UPOLL086-09† | *Halictus ligatus* | Cambridge, ON, CA | Hl06 |
| UPOLL087-09† | *Halictus ligatus* | Cambridge, ON, CA | Hl06 |
| UPOLL088-09† | *Halictus ligatus* | Cambridge, ON, CA | Hl06 |
| UPOLL090-09† | *Halictus ligatus* | Cambridge, ON, CA | Hl06 |
| UPOLL095-09† | *Halictus ligatus* | Cambridge, ON, CA | Hl06 |
| UPOLL097-09† | *Halictus ligatus* | Cambridge, ON, CA | Hl06 |
| UPOLL100-09† | *Halictus ligatus* | Cambridge, ON, CA | Hl06 |
| UPOLL101-09† | *Halictus ligatus* | Cambridge, ON, CA | Hl06 |
| UPOLL103-09† | *Halictus ligatus* | Cambridge, ON, CA | Hl06 |
| UPOLL105-09† | *Halictus ligatus* | Cambridge, ON, CA | Hl06 |
| UPOLL107-09† | *Halictus ligatus* | Cambridge, ON, CA | Hl06 |
| UPOLL115-09† | *Halictus ligatus* | Cambridge, ON, CA | Hl06 |
| UPOLL160-09† | *Halictus ligatus* | Cambridge, ON, CA | Hl06 |
| UPOLL167-09† | *Halictus ligatus* | Cambridge, ON, CA | Hl06 |
| UPOLL170-09† | *Halictus ligatus* | Cambridge, ON, CA | Hl06 |
| UPOLL175-09† | *Halictus ligatus* | Cambridge, ON, CA | Hl06 |
| UPOLL228-09† | *Halictus ligatus* | Cambridge, ON, CA | Hl06 |
| UPOLL232-09† | *Halictus ligatus* | Cambridge, ON, CA | Hl06 |
| UPOLL238-09† | *Halictus ligatus* | Cambridge, ON, CA | Hl06 |
| UPOLL242-09† | *Halictus ligatus* | Cambridge, ON, CA | Hl06 |
| UPOLL245-09† | *Halictus ligatus* | Cambridge, ON, CA | Hl06 |
| UPOLL248-09† | *Halictus ligatus* | Cambridge, ON, CA | Hl06 |
| UPOLL278-09† | *Halictus ligatus* | Cambridge, ON, CA | Hl06 |
| UPOLL279-09† | *Halictus ligatus* | Cambridge, ON, CA | Hl06 |
| UPOLL281-09† | *Halictus ligatus* | Cambridge, ON, CA | Hl06 |
| UPOLL282-09† | *Halictus ligatus* | Cambridge, ON, CA | Hl06 |
| UPOLL283-09† | *Halictus ligatus* | Cambridge, ON, CA | Hl06 |
| UPOLL351-09† | *Halictus ligatus* | Cambridge, ON, CA | Hl06 |
| UPOLL382-09† | *Halictus ligatus* | Guelph, ON, CA | Hl06 |
| UPOLL395-09† | *Halictus ligatus* | Guelph, ON, CA | Hl06 |
| UPOLL411-09† | *Halictus ligatus* | Guelph, ON, CA | Hl06 |
| UPOLL413-09† | *Halictus ligatus* | Guelph, ON, CA | Hl06 |
| UPOLL436-09† | *Halictus ligatus* | Cambridge, ON, CA | Hl06 |
| UPOLL438-09† | *Halictus ligatus* | Cambridge, ON, CA | Hl06 |
| UPOLL440-09† | *Halictus ligatus* | Cambridge, ON, CA | Hl06 |
| UPOLL441-09† | *Halictus ligatus* | Cambridge, ON, CA | Hl06 |
| UPOLL443-09† | *Halictus ligatus* | Cambridge, ON, CA | Hl06 |
| UPOLL446-09† | *Halictus ligatus* | Cambridge, ON, CA | Hl06 |
| UPOLL447-09† | *Halictus ligatus* | Cambridge, ON, CA | Hl06 |
| UPOLL449-09† | *Halictus ligatus* | Cambridge, ON, CA | Hl06 |
| UPOLL453-09† | *Halictus ligatus* | Cambridge, ON, CA | Hl06 |
| UPOLL457-09† | *Halictus ligatus* | Cambridge, ON, CA | Hl06 |
| UPOLL460-09† | *Halictus ligatus* | Cambridge, ON, CA | Hl06 |
| UPOLL461-09† | *Halictus ligatus* | Cambridge, ON, CA | Hl06 |
| UPOLL462-09† | *Halictus ligatus* | Cambridge, ON, CA | Hl06 |
| UPOLL463-09† | *Halictus ligatus* | Cambridge, ON, CA | Hl06 |
| UPOLL464-09† | *Halictus ligatus* | Cambridge, ON, CA | Hl06 |
| UPOLL465-09† | *Halictus ligatus* | Cambridge, ON, CA | Hl06 |
| UPOLL468-09† | *Halictus ligatus* | Cambridge, ON, CA | Hl06 |
| UPOLL470-09† | *Halictus ligatus* | Cambridge, ON, CA | Hl06 |
| UPOLL473-09† | *Halictus ligatus* | Cambridge, ON, CA | Hl06 |
| UPOLL474-09† | *Halictus ligatus* | Cambridge, ON, CA | Hl06 |
| UPOLL475-09† | *Halictus ligatus* | Cambridge, ON, CA | Hl06 |
| UPOLL477-09† | *Halictus ligatus* | Cambridge, ON, CA | Hl06 |
| UPOLL480-09† | *Halictus ligatus* | Cambridge, ON, CA | Hl06 |
| UPOLL486-09† | *Halictus ligatus* | Cambridge, ON, CA | Hl06 |
| UPOLL487-09† | *Halictus ligatus* | Cambridge, ON, CA | Hl06 |
| UPOLL491-09† | *Halictus ligatus* | Cambridge, ON, CA | Hl06 |
| UPOLL494-09† | *Halictus ligatus* | Cambridge, ON, CA | Hl06 |
| UPOLL495-09† | *Halictus ligatus* | Cambridge, ON, CA | Hl06 |
| UPOLL509-09† | *Halictus ligatus* | Guelph, ON, CA | Hl06 |
| UPOLL517-09† | *Halictus ligatus* | Guelph, ON, CA | Hl06 |
| UPOLL537-09† | *Halictus ligatus* | Guelph, ON, CA | Hl06 |
| UPOLL545-09† | *Halictus ligatus* | Guelph, ON, CA | Hl06 |
| UPOLL560-09† | *Halictus ligatus* | Cambridge, ON, CA | Hl06 |
| UPOLL578-09† | *Halictus ligatus* | Cambridge, ON, CA | Hl06 |
| UPOLL583-09† | *Halictus ligatus* | Cambridge, ON, CA | Hl06 |
| UPOLL584-09† | *Halictus ligatus* | Cambridge, ON, CA | Hl06 |
| UPOLL588-09† | *Halictus ligatus* | Cambridge, ON, CA | Hl06 |
| UPOLL589-09† | *Halictus ligatus* | Cambridge, ON, CA | Hl06 |
| UPOLL591-09† | *Halictus ligatus* | Cambridge, ON, CA | Hl06 |
| UPOLL596-09† | *Halictus ligatus* | Cambridge, ON, CA | Hl06 |
| UPOLL597-09† | *Halictus ligatus* | Cambridge, ON, CA | Hl06 |
| UPOLL599-09† | *Halictus ligatus* | Cambridge, ON, CA | Hl06 |
| UPOLL602-09† | *Halictus ligatus* | Cambridge, ON, CA | Hl06 |
| UPOLL604-09† | *Halictus ligatus* | Cambridge, ON, CA | Hl06 |
| UPOLL606-09† | *Halictus ligatus* | Cambridge, ON, CA | Hl06 |
| UPOLL608-09† | *Halictus ligatus* | Cambridge, ON, CA | Hl06 |
| UPOLL611-09† | *Halictus ligatus* | Cambridge, ON, CA | Hl06 |
| UPOLL614-09† | *Halictus ligatus* | Cambridge, ON, CA | Hl06 |
| UPOLL617-09† | *Halictus ligatus* | Cambridge, ON, CA | Hl06 |
| UPOLL632-09† | *Halictus ligatus* | Guelph, ON, CA | Hl06 |
| UPOLL649-09† | *Halictus ligatus* | Guelph, ON, CA | Hl06 |
| UPOLL661-09† | *Halictus ligatus* | Cambridge, ON, CA | Hl06 |
| UPOLL667-09† | *Halictus ligatus* | Cambridge, ON, CA | Hl06 |
| UPOLL668-09† | *Halictus ligatus* | Cambridge, ON, CA | Hl06 |
| UPOLL673-09† | *Halictus ligatus* | Cambridge, ON, CA | Hl06 |
| UPOLL677-09† | *Halictus ligatus* | Cambridge, ON, CA | Hl06 |
| UPOLL668-09† | *Halictus ligatus* | Cambridge, ON, CA | Hl06 |
| UPOLL673-09† | *Halictus ligatus* | Cambridge, ON, CA | Hl06 |
| UPOLL677-09† | *Halictus ligatus* | Guelph, ON, CA | Hl06 |
| UPOLL678-09† | *Halictus ligatus* | Guelph, ON, CA | Hl06 |
| UPOLL679-09† | *Halictus ligatus* | Guelph, ON, CA | Hl06 |
| UPOLL712-09† | *Halictus ligatus* | Guelph, ON, CA | Hl06 |
| UPOLL714-09† | *Halictus ligatus* | Guelph, ON, CA | Hl06 |
| UPOLL716-09† | *Halictus ligatus* | Guelph, ON, CA | Hl06 |
| UPOLL719-09† | *Halictus ligatus* | Guelph, ON, CA | Hl06 |
| UPOLL720-09† | *Halictus ligatus* | Guelph, ON, CA | Hl06 |
| UPOLL743-09† | *Halictus ligatus* | Guelph, ON, CA | Hl06 |
| UPOLL753-09† | *Halictus ligatus* | Cambridge, ON, CA | Hl06 |
| UPOLL755-09† | *Halictus ligatus* | Cambridge, ON, CA | Hl06 |
| UPOLL756-09† | *Halictus ligatus* | Cambridge, ON, CA | Hl06 |
| UPOLL757-09† | *Halictus ligatus* | Cambridge, ON, CA | Hl06 |
| UPOLL762-09† | *Halictus ligatus* | Cambridge, ON, CA | Hl06 |
| UPOLL794-09† | *Halictus ligatus* | Guelph, ON, CA | Hl06 |
| UPOLL835-09† | *Halictus ligatus* | Guelph, ON, CA | Hl06 |
| UPOLL836-09† | *Halictus ligatus* | Guelph, ON, CA | Hl06 |
| UPOLL837-09† | *Halictus ligatus* | Guelph, ON, CA | Hl06 |
| UPOLL839-09† | *Halictus ligatus* | Guelph, ON, CA | Hl06 |
| UPOLL853-09† | *Halictus ligatus* | Cambridge, ON, CA | Hl06 |
| UPOLL860-09† | *Halictus ligatus* | Guelph, ON, CA | Hl06 |
| UPOLL861-09† | *Halictus ligatus* | Guelph, ON, CA | Hl06 |
| UPOLL868-09† | *Halictus ligatus* | Cambridge, ON, CA | Hl06 |
| UPOLL871-09† | *Halictus ligatus* | Cambridge, ON, CA | Hl06 |
| UPOLL872-09† | *Halictus ligatus* | Cambridge, ON, CA | Hl06 |
| UPOLL877-09† | *Halictus ligatus* | Cambridge, ON, CA | Hl06 |
| UPOLL878-09† | *Halictus ligatus* | Cambridge, ON, CA | Hl06 |
| UPOLL892-09† | *Halictus ligatus* | Guelph, ON, CA | Hl06 |
| UPOLL921-09† | *Halictus ligatus* | Cambridge, ON, CA | Hl06 |
| UPOLL922-09† | *Halictus ligatus* | Cambridge, ON, CA | Hl06 |
| UPOLL927-09† | *Halictus ligatus* | Guelph, ON, CA | Hl06 |
| UPOLL935-09† | *Halictus ligatus* | Guelph, ON, CA | Hl06 |
| UPOLL940-09† | *Halictus ligatus* | Waterloo, ON, CA | Hl06 |
| BOWGF1918-10† | *Halictus ligatus* | York University, ON, CA | Hl07 |
| BUSA174-05† | *Halictus ligatus* | Frederick, VA, USA | Hl07 |
| MN022* | *Halictus ligatus* | Mountain Research Station, NC, USA | Hl07 |
| OPPOI291-17† | *Halictus ligatus* | Warsaw, ON, CA | Hl07 |
| UPOLB086-09† | *Halictus ligatus* | Cambridge, ON, CA | Hl07 |
| UPOLB128-09† | *Halictus ligatus* | Kitchener, ON, CA | Hl07 |
| UPOLB162-09† | *Halictus ligatus* | Cambridge, ON, CA | Hl07 |
| UPOLB265-09† | *Halictus ligatus* | Cambridge, ON, CA | Hl07 |
| UPOLB421-09† | *Halictus ligatus* | Cambridge, ON, CA | Hl07 |
| UPOLB721-09† | *Halictus ligatus* | Guelph, ON, CA | Hl07 |
| UPOLL006-09† | *Halictus ligatus* | Cambridge, ON, CA | Hl07 |
| UPOLL012-09† | *Halictus ligatus* | Cambridge, ON, CA | Hl07 |
| UPOLL013-09† | *Halictus ligatus* | Cambridge, ON, CA | Hl07 |
| UPOLL053-09† | *Halictus ligatus* | Cambridge, ON, CA | Hl07 |
| UPOLL054-09† | *Halictus ligatus* | Cambridge, ON, CA | Hl07 |
| UPOLL080-09† | *Halictus ligatus* | Cambridge, ON, CA | Hl07 |
| UPOLL092-09† | *Halictus ligatus* | Cambridge, ON, CA | Hl07 |
| UPOLL093-09† | *Halictus ligatus* | Cambridge, ON, CA | Hl07 |
| UPOLL102-09† | *Halictus ligatus* | Cambridge, ON, CA | Hl07 |
| UPOLL118-09† | *Halictus ligatus* | Cambridge, ON, CA | Hl07 |
| UPOLL237-09† | *Halictus ligatus* | Cambridge, ON, CA | Hl07 |
| UPOLL244-09† | *Halictus ligatus* | Cambridge, ON, CA | Hl07 |
| UPOLL246-09† | *Halictus ligatus* | Cambridge, ON, CA | Hl07 |
| UPOLL247-09† | *Halictus ligatus* | Cambridge, ON, CA | Hl07 |
| UPOLL285-09† | *Halictus ligatus* | Cambridge, ON, CA | Hl07 |
| UPOLL403-09† | *Halictus ligatus* | Guelph, ON, CA | Hl07 |
| UPOLL404-09† | *Halictus ligatus* | Guelph, ON, CA | Hl07 |
| UPOLL412-09† | *Halictus ligatus* | Guelph, ON, CA | Hl07 |
| UPOLL445-09† | *Halictus ligatus* | Cambridge, ON, CA | Hl07 |
| UPOLL454-09† | *Halictus ligatus* | Cambridge, ON, CA | Hl07 |
| UPOLL469-09† | *Halictus ligatus* | Cambridge, ON, CA | Hl07 |
| UPOLL482-09† | *Halictus ligatus* | Cambridge, ON, CA | Hl07 |
| UPOLL580-09† | *Halictus ligatus* | Cambridge, ON, CA | Hl07 |
| UPOLL658-09† | *Halictus ligatus* | Cambridge, ON, CA | Hl07 |
| UPOLL670-09† | *Halictus ligatus* | Cambridge, ON, CA | Hl07 |
| UPOLL671-09† | *Halictus ligatus* | Cambridge, ON, CA | Hl07 |
| UPOLL741-09† | *Halictus ligatus* | Cambridge, ON, CA | Hl07 |
| UPOLL758-09† | *Halictus ligatus* | Cambridge, ON, CA | Hl07 |
| UPOLL774-09† | *Halictus ligatus* | Cambridge, ON, CA | Hl07 |
| UPOLL805-09† | *Halictus ligatus* | Guelph, ON, CA | Hl07 |
| UPOLL838-09† | *Halictus ligatus* | Cambridge, ON, CA | Hl07 |
| UPOLL903-09† | *Halictus ligatus* | Guelph, ON, CA | Hl07 |
| UPOLB436-09† | *Halictus ligatus* | Guelph, ON, CA | Hl08 |
| UPOLL196-09† | *Halictus ligatus* | Guelph, ON, CA | Hl08 |
| UPOLB153-09† | *Halictus ligatus* | Cambridge, ON, CA | Hl09 |
| UPOLB232-09† | *Halictus ligatus* | Guelph, ON, CA | Hl09 |
| UPOLL600-09† | *Halictus ligatus* | Cambridge, ON, CA | Hl09 |
| UPOLL616-09† | *Halictus ligatus* | Cambridge, ON, CA | Hl09 |
| UPOLL949-09† | *Halictus ligatus* | Cambridge, ON, CA | Hl09 |
| UPOLB166-09† | *Halictus ligatus* | Cambridge, ON, CA | Hl10 |
| UPOLL104-09† | *Halictus ligatus* | Cambridge, ON, CA | Hl10 |
| UPOLL459-09† | *Halictus ligatus* | Cambridge, ON, CA | Hl10 |
| UPOLL579-09† | *Halictus ligatus* | Cambridge, ON, CA | Hl10 |
| UPOLL717-09† | *Halictus ligatus* | Guelph, ON, CA | Hl10 |
| UPOLL763-09† | *Halictus ligatus* | Cambridge, ON, CA | Hl10 |
| JSHYN469-11† | *Halictus ligatus* | Leeds and Grenville, ON, CA | Hl11 |
| JSHYN624-11† | *Halictus ligatus* | Leeds and Grenville, ON, CA | Hl11 |
| JSHYN677-11† | *Halictus ligatus* | Leeds and Grenville, ON, CA | Hl11 |
| JSHYO471-11† | *Halictus ligatus* | Leeds and Grenville, ON, CA | Hl11 |
| JSHYO925-11† | *Halictus ligatus* | Leeds and Grenville, ON, CA | Hl11 |
| JSHYP121-11† | *Halictus ligatus* | Leeds and Grenville, ON, CA | Hl11 |
| NCCC649-11† | *Halictus ligatus* | Scheck Nature Reserve, ON, CA | Hl11 |
| NCCC650-11† | *Halictus ligatus* | Scheck Nature Reserve, ON, CA | Hl11 |
| NCCC652-11† | *Halictus ligatus* | Scheck Nature Reserve, ON, CA | Hl11 |
| OPPOI297-17† | *Halictus ligatus* | Warsaw, ON, CA | Hl11 |
| UPOLB108-09† | *Halictus ligatus* | Cambridge, ON, CA | Hl11 |
| UPOLL009-09† | *Halictus ligatus* | Cambridge, ON, CA | Hl11 |
| UPOLL410-09† | *Halictus ligatus* | Guelph, ON, CA | Hl11 |
| UPOLL557-09† | *Halictus ligatus* | Guelph, ON, CA | Hl11 |
| UPOLL603-09† | *Halictus ligatus* | Cambridge, ON, CA | Hl11 |
| UPOLL901-09† | *Halictus ligatus* | Guelph, ON, CA | Hl11 |
| UPOLB154-09† | *Halictus ligatus* | Cambridge, ON, CA | Hl12 |
| UPOLL042-09† | *Halictus ligatus* | Cambridge, ON, CA | Hl12 |
| UPOLL055-09† | *Halictus ligatus* | Cambridge, ON, CA | Hl12 |
| UPOLL585-09† | *Halictus ligatus* | Cambridge, ON, CA | Hl12 |
| BEEMH297-06† | *Halictus ligatus* | Guelph, ON, CA | Hl13 |
| OPPOE463-17† | *Halictus ligatus* | Warsaw, ON, CA | Hl13 |
| UPOLB031-09† | *Halictus ligatus* | Cambridge, ON, CA | Hl13 |
| UPOLL033-09† | *Halictus ligatus* | Waterloo, ON, CA | Hl13 |
| UPOLL056-09† | *Halictus ligatus* | Cambridge, ON, CA | Hl13 |
| UPOLL066-09† | *Halictus ligatus* | Cambridge, ON, CA | Hl13 |
| UPOLL078-09† | *Halictus ligatus* | Cambridge, ON, CA | Hl13 |
| UPOLL094-09† | *Halictus ligatus* | Cambridge, ON, CA | Hl13 |
| UPOLL119-09† | *Halictus ligatus* | Cambridge, ON, CA | Hl13 |
| UPOLL197-09† | *Halictus ligatus* | Guelph, ON, CA | Hl13 |
| UPOLL396-09† | *Halictus ligatus* | Guelph, ON, CA | Hl13 |
| UPOLL455-09† | *Halictus ligatus* | Cambridge, ON, CA | Hl13 |
| UPOLL534-09† | *Halictus ligatus* | Guelph, ON, CA | Hl13 |
| UPOLL542-09† | *Halictus ligatus* | Guelph, ON, CA | Hl13 |
| UPOLL559-09† | *Halictus ligatus* | Guelph, ON, CA | Hl13 |
| UPOLL595-09† | *Halictus ligatus* | Cambridge, ON, CA | Hl13 |
| UPOLL620-09† | *Halictus ligatus* | Cambridge, ON, CA | Hl13 |
| UPOLL674-09† | *Halictus ligatus* | Cambridge, ON, CA | Hl13 |
| UPOLL685-09† | *Halictus ligatus* | Guelph, ON, CA | Hl13 |
| UPOLL744-09† | *Halictus ligatus* | Guelph, ON, CA | Hl13 |
| UPOLL790-09† | *Halictus ligatus* | Guelph, ON, CA | Hl13 |
| UPOLL793-09† | *Halictus ligatus* | Guelph, ON, CA | Hl13 |
| UPOLL842-09† | *Halictus ligatus* | Guelph, ON, CA | Hl13 |
| UPOLL904-09† | *Halictus ligatus* | Guelph, ON, CA | Hl13 |
| UPOLL931-09† | *Halictus ligatus* | Guelph, ON, CA | Hl13 |
| UPOLL934-09† | *Halictus ligatus* | Guelph, ON, CA | Hl13 |
| PIMA168-19† | *Halictus ligatus* | Tucson, AZ, USA | Hl14 |
| UPOLL847-09† | *Halictus ligatus* | Kitchener, ON, CA | Hl15 |
| UPOLB094-09† | *Halictus ligatus* | Guelph, ON, CA | Hl16 |
| UAIC056-20† | *Halictus ligatus* | Cochise County, AZ, USA | Hl17 |
| BEEMH120-06† | *Halictus ligatus* | Guelph, ON, CA | Hl18 |
| TDWGB023-10† | *Halictus ligatus* | Barnstable County, MA, USA | Hl19 |
| OPPOE290-17† | *Halictus ligatus* | Warsaw, ON, CA | Hl20 |
| UPOLL205-09† | *Halictus ligatus* | Kitchener, ON, CA | Hl21 |
| UPOLL489-09† | *Halictus ligatus* | Cambridge, ON, CA | Hl22 |
| UPOLB003-09† | *Halictus ligatus* | Cambridge, ON, CA | Hl23 |
| MPGF1128-19† | *Halictus ligatus* | Florence, MT, USA | Hl24 |
| BUSA177-05† | *Halictus ligatus* | Anne Arundel, MD, USA | Hl25 |
| MPGS914-19† | *Halictus ligatus* | Florence, MT, USA | Hl26 |
| BBHYA963-12 | *Halictus* sp. | Bentsen-Rio Grande Valley State Park, TX, USA | TX04 |
| BBHYA968-12 | *Halictus* sp. | Bentsen-Rio Grande Valley State Park, TX, USA | TX05 |
| BBHYA969-12 | *Halictus* sp. | Bentsen-Rio Grande Valley State Park, TX, USA | TX01 |
| BBHYA971-12 | *Halictus* sp. | Bentsen-Rio Grande Valley State Park, TX, USA | TX03 |
| BBHYA972-12 | *Halictus* sp. | Bentsen-Rio Grande Valley State Park, TX, USA | TX01 |
| BBHYA1325-12 | *Halictus* sp. | Bentsen-Rio Grande Valley State Park, TX, USA | TX02 |
| BOFWM051-08 | *Halictus townsendi* | Acatapec, Puebla, MX | Ht01 |
| BOFWM151-08 | *Halictus townsendi* | San Sebastien de la Frontera, Oaxaca, MX | Ht01 |
| BOFWM152-08 | *Halictus townsendi* | San Sebastien de la Frontera, Oaxaca, MX | Ht01 |
| BOTWA437-11 | *Halictus townsendi* | San Sebastien de la Frontera, Oaxaca, MX | Ht06 |
| BOTWA441-11 | *Halictus townsendi* | Acatapec, Puebla, MX | Ht01 |
| BOTWA444-11 | *Halictus townsendi* | Acatapec, Puebla, MX | Ht01 |
| BOWGF1530-10 | *Halictus townsendi* | Sta. Lucia Lachua, Coban, Alta Verapaz, GT | Ht02 |
| BOWGF1540-10 | *Halictus townsendi* | Sta. Lucia Lachua, Coban, Alta Verapaz, GT | Ht02 |
| HMBME148-07 | *Halictus townsendi* | Jardin Botanico De Zapotitlan Salinas, Puebla, MX | Ht01 |
| NTCOL008-15 | *Halictus townsendi* | Rancho los Cortex, Jalisco, MX | Ht03 |
| UAIC1555-21 | *Halictus townsendi* | San Carlos, Guaymas, Sonora, MX | Ht04 |
| UAIC357-21 | *Halictus townsendi* | San Carlos, Guaymas, Sonora, MX | Ht05 |
| AF102842‡ | *Halictus rubicundus* | Missoula, MT, USA |  |
| JQ266430‡ | *Halictus rubicundus* | Missoula, MT, USA |  |
| JQ266431‡ | *Halictus rubicundus* | Moore County, NC, USA |  |
| KT164668‡ | *Halictus rubicundus* | Chilterns National Landscape, EN, UK |  |
| BB408* | *Lasioglossum lustrans* | Border Belt Research Station, NC, USA |  |

Appendix 7. A neighbor-joining tree of CO1 sequence data generated from combined data from this study and public data mined from BOLD for *H. ligatus*, *H. poeyi*, *H. townsendi*, and *Halictus* sp. from Texas. *Lasioglossum lustrans* is included as an outgroup.
